# Revealing the Oxygen Transport Challenges in Catalyst Layers in Proton Exchange Membrane Fuel Cells and Water Electrolysis

**DOI:** 10.1007/s40820-025-01719-y

**Published:** 2025-04-21

**Authors:** Huiyuan Li, Shu Yuan, Jiabin You, Congfan Zhao, Xiaojing Cheng, Liuxuan Luo, Xiaohui Yan, Shuiyun Shen, Junliang Zhang

**Affiliations:** 1 Institute of Fuel Cells, School of Mechanical Engineering, Shanghai Jiao Tong University, Shanghai, 200240 People’s Republic of China; 2 MOE Key Laboratory of Power & Machinery Engineering, Shanghai Jiao Tong University, Shanghai, 200240 People’s Republic of China

**Keywords:** Proton exchange membrane fuel cells, Water electrolysis, Oxygen transport, Pore structure, Ionomer thin films, Agglomerate engineering

## Abstract

Urgent requirements of the renewable energy boost the development of stable and clean hydrogen, which could effectively displace fossil fuels in mitigating climate changes. The efficient interconversion of hydrogen and electronic is highly based on polymer electrolyte membrane fuel cells (PEMFCs) and water electrolysis (PEMWEs). However, the high cost continues to impede large-scale commercialization of both PEMFC and PEMWE technologies, with the expense primarily attributed to noble catalysts serving as a major bottleneck. The reduction of Pt loading in PEMFCs is essential but limited by the oxygen transport resistance in the cathode catalyst layers (CCLs), while the oxygen transport in anode catalyst layers (ACLs) in PEMWEs also being focused as the Ir/IrO_*x*_ catalyst reduced. The pore structure and the catalyst–ionomer agglomerates play important roles in the oxygen transport process of both PEMFCs and PEMWEs due to the similarity of membrane electrode assembly (MEA). Herein, the oxygen transport mechanism of PEMFCs in pore structure and ionomer thin films in CCLs is systematically reviewed, while state-of-the-art strategies are presented for enhancing oxygen transport and performance through materials and structural design. The deeply research opens avenues for exploring similar key scientific problems in oxygen transport process of PEMWEs and their further development.

## Highlights

- Mechanisms of the bulk and local oxygen transport in cathode catalyst layers (CCLs) in proton exchange membrane fuel cells (PEMFCs) are presented.
- State-of-the-art strategies to mitigate the oxygen transport resistance in CCLs in PEMFCs are reviewed, including the novel structure design, carbon supports design, and ionomer design.
- New directions for oxygen transport development in anode catalyst layers (ACLs) in proton exchange membrane water electrolysis (PEMWEs) are inspired by the PEMFCs.

## Graphical abstract

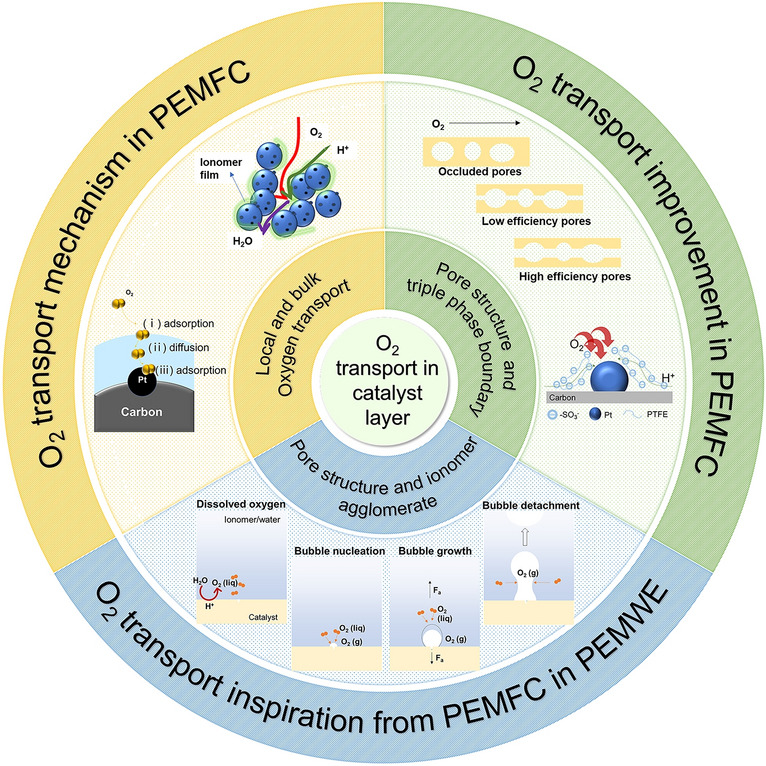

## Introduction

To meet the energy demand without fossil fuels and accelerating greenhouse gas emissions, it is imperative to increase uptake of clean energy. Hydrogen offers a broad range of benefits, including high calorific value, which is 2–3 times that of petroleum, stable energy output, wide range of sources and zero pollution [1]. It is believed that the decarbonization of the global economy in the upcoming years would heavily rely on the pivotal role of hydrogen. The entire “hydrogen-electrode” industry chain aims to achieve optimal utilization of hydrogen, encompassing upstream production, midstream storage, and downstream usage [2].

The fuel cell, which finds widespread applications in stationary, transportation, and portable sectors, is considered to be one of the most rapidly advancing and well-established forms of hydrogen utilization. It is regarded as a promising power device that directly converts the chemical energy stored in hydrogen to electrical energy through electrochemical reactions, including alkaline fuel cell (AFC), phosphoric acid fuel cell (PAFC), molten carbonate fuel cell (MCFC), solid oxide fuel cell (SOFC), and proton exchange membrane fuel cell (PEMFC). Among them, PEMFCs are receiving the most attention especially for transportation and portable charging because of their highly efficient energy conversion, low operating temperature, and strong reliability [3, 4].

On the other hand, water electrolysis represents a reverse process for generating hydrogen from water through electrolytic reactions, providing an environmentally friendly alternative approach for producing green hydrogen. The primary advantage of green hydrogen lies in its ability to enable maximum utilization of electrical energy, thereby optimizing the use of low-quality electrical sources such as solar, wind, or even nuclear power, enhancing compatibility with dynamic current loads, and balancing the volatility when integrating renewable energy resources. With the significant increase in the installed capacity of renewable energy, the production cost of green hydrogen will be further reduced, and hydrogen production technology from renewable energy will gradually become the mainstream. Water electrolysis devices could be divided into alkaline liquid water electrolysis (ALWEs), alkaline membrane water electrolysis (AMWEs), proton exchange membrane water electrolysis (PEMWEs), and solid oxide water electrolysis (SOECs). The high efficiency, low maintenance cost, fast dynamics, and high hydrogen purity of PEMWEs have garnered increasing attention [5].

**Fig. 1:**
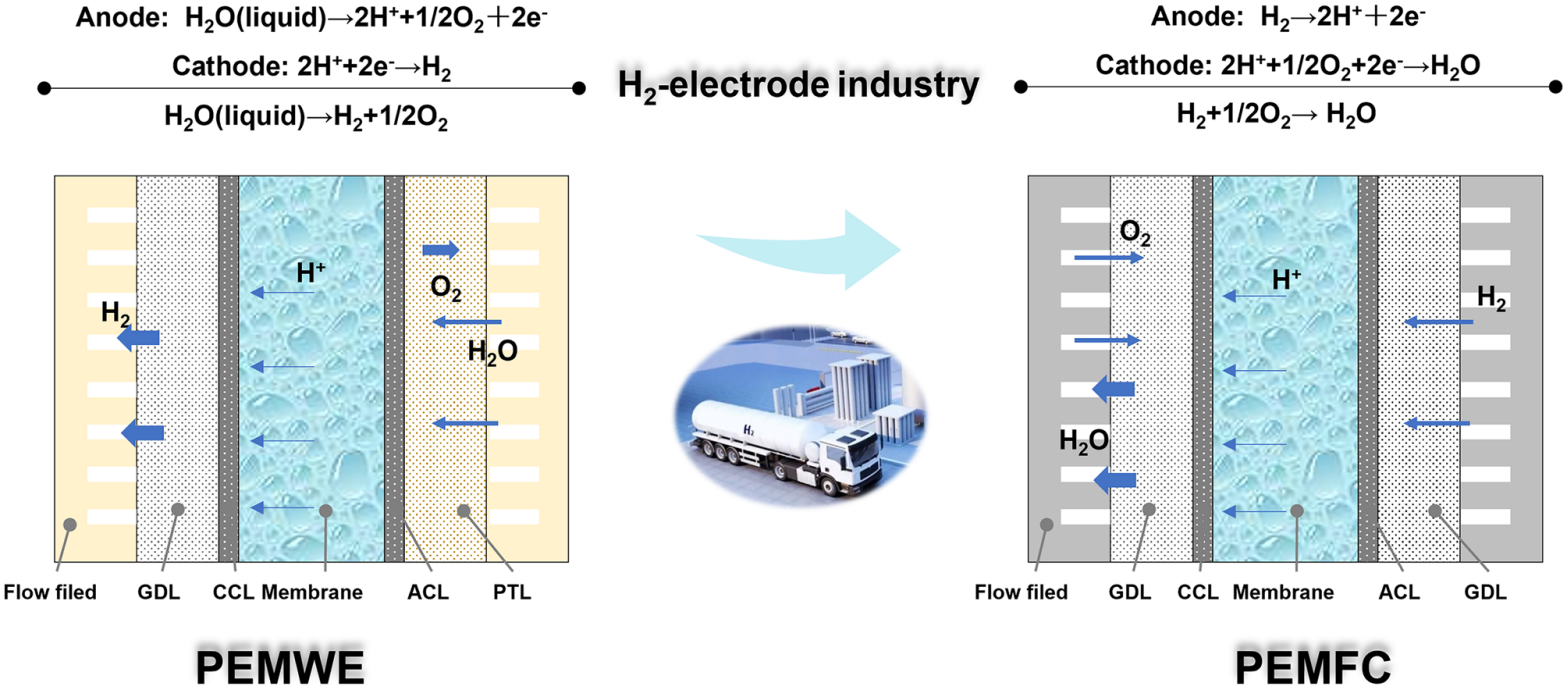
Schematics of the PEMWE (left) and PEMWE (right). Operation principles, reactions, and structures [2, 6]

PEMFCs and PEMWEs share a similar structure, consisting of a proton exchange membrane (PEM) with catalyst layers (CLs) coated on its surfaces, gas diffusion layer (GDL) or porous transport layer (PTL), flow fields, and bipolar plates (BPs), from the core membrane electrode assembly (MEA) of a single cell to the outside as shown in Fig. 1 [2, 6]. Both systems work at low temperatures (e.g., 60–80 °C) and can operate free of CO_2_ emissions. The operation of PEMWEs is characterized by the reverse process of PEMFCs, thus providing a promising solution for renewable hydrogen supply in fueling infrastructure [6, 7]. The PEMFC and WE devices are complementary and sustainable technologies. Their integration facilitates energy recycling, thereby contributing to the advancement of clean energy systems, such as the development of fuel cell electrode vehicles (FCEVs), distributed energy systems (DES), and the unitized regenerative fuel cells (URFCs) [8]. For example, the rapid development of PEMFCs in FCEVs has become competitive in markets, as the companies like Toyota, Honda, and Hyundai have recently launched their Mirai, Clarity, and Tuscon. The WE could then provide renewable energy-based fuel for hydrogen refueling stations and support the promotion of FCEVs.

However, the cost and durability still limit their high performance and large-scale commercialization in PEMFC. To reach the US DOE ultimate target, the PEMFC cost for light-duty vehicles needs to be reduced below 30 $ kW^−1^ and for heavy-duty vehicles needs to be reduced below  60 $ kW^−1^ [9]. The catalyst for PEMFCs utilized in the cathode to facilitate slow kinetics oxygen reduction reaction (ORR) is responsible for approximately 41% of the cost of fuel cell stacks when producing an annual output of 500,000 sets [4, 9].

Tremendous attempts have been focused on exploiting catalyst with low cost, outstanding activity, and long stability for PEMFCs to decrease the use of the scarcity of Pt catalysts [10, 11]. Pt alloy-based materials (i.e., Pt–Co, Pt–Ni, and Pt–Fe alloys) demonstrate outstanding activity due to the shifts in the d-band center with the Pt alloy composition leading to altered binding strength of oxygenated species [12]. Further, to make the active center of the catalyst more easily exposed and improve the utilization, the dramatic facet effect has inspired many researchers to design and prepare catalysts with morphology control [13], such as ordered intermetallic structure [14, 15] and nitrogen-doped core–shell catalysts [16]. The interaction between Pt shells and core substrates can tune the electronic state of the surface Pt, and the outer Pt shell with modest thickness can enhance the oxidation and dissolution resistance of the core [17]. However, there is still a large gap between the ultra-low Pt loading of 0.1 g kW^−1^ and the long-term sustainable target of 5 g per vehicle for FCEVs [18], which represents the commercial level to compete with the internal combustion engine in practical applications. The high mass activity and specific activity of catalysts based on well-established methodologies have obtained yet from rotating disk electrode (RDE) measurements, while the performance and stability of the catalyst have rarely been effectively translated into MEA-level [19]. The MEA consisting of cathode catalyst layer (CCL), anode catalyst layer (ACL), and proton exchange membrane (PEM) plays a crucial role in determining both the kinetics and mass transport, thereby significantly impacting the performance of PEMFCs. Nafion was firstly developed by Dupont in 1970s as a chemically stable PEM based on sulfonated polytetrafluoroethylene, leading to a large-scale use of this membrane in the energy storage or conversion systems. Currently, the prevailing trend in PEM development is toward thinner membranes to decrease the distance for proton conduction, while simultaneously ensuring the preservation of their mechanical properties [20]. On the side, the maintenance of a highly active CL structure is more challenging in MEAs because of the harsher reaction conditions, including higher operating currents and temperatures, and more frequent voltage changes during automotive applications.

In an RDE measurement, the CL on the glassy carbon rotating electrode exhibits a thin-film structure with two-phase interfaces (liquid electrolyte and catalyst) as the active sites for reactions. The dissolved oxygen and protons in the liquid electrolyte are supplied through rotation of the electrode, while the CL in a MEA is about six to ten times thicker than RDE-level and the reaction zones are the well-known triple-phase boundaries (TPBs) including the catalyst for conducting electrons, ionomer for conducting protons and pores for transporting gases or water [21]. The well-known poisoning effect caused by the adsorption of the perfluorosulfonic acid (PFSA) ionomer’s sulfonic groups (–SO_3_^−^) on the Pt surface is more pronounced in the CL of an MEA than in an RDE because the affinity of –SO_3_^−^ groups for the Pt surface could be weaker due to the stronger hydrophilicity of aqueous HClO_4_ electrolyte in RDE conditions. What’s more, the mass transport in MEA should be more considerable than in RDE due to complex porous structure, multiple components, and their interfacial relations. In particular, the transport of oxygen, starting from channels and then passing through pores in GDLs and CCLs, before finally crossing a thin ionomer film, poses a significantly greater obstacle compared to the RDE conditions. The GDL plays multifaceted roles in cell operation by controlling mass, heat, and electron transport with robust mechanical support during operation. Wettability and pore structure of GDL are important factors affecting its water and gas transport, as an ideal GDL would require fast removal of liquid water, and provide smooth gas transport channel. Due to the complex nanoscales in TPBs, the oxygen transport process in the CLs becomes increasingly important when considering low or ultra-low Pt loading MEAs [22, 23], and this review thus will focus on the transport in CLs.

For PEMWEs, the reduction loading of carbon-supported Pt used for hydrogen evolution reaction (HER) in the CCL has shown to be possible without any impact on performance due to the extremely fast HER kinetics of Pt in acidic electrolytes [24, 25]. However, due to the highly corrosive and acidic operating environment in the ACL, the precious metal catalyst iridium or iridium oxide (Ir/IrO_*x*_)-based catalysts are the only feasible anode catalyst yet for high durability and activity oxygen evolution reaction (OER) [26]. It is very similar to the PEMFCs that the high loadings of the anode catalyst materials are expected to become a major cost contributor in PEMWE systems, as the contribution of balance-of-plant costs and other stack components will be much lower for larger systems (MW range) [27]. The oxygen transport direction is opposite to that in PEMFC, where the oxygen will act as the production on the active sites and then remove through the ACL, PTL, and flow field as fast as possible. Bubbles will form when the amount of oxygen produced increases to the saturation, and then impede the transport of reactant water, causing serious transport problems, and reducing the efficiency of PEMWEs. Similar to PEMFCs, the structure of the ACL in PEMWEs is also composed of ionomer except for the catalyst, while the difference is that the catalyst particle size is relatively small. The mechanism of oxygen transport in PEMWEs has not been fully understood, and further research is urgently needed.

Furthermore, the durability of the hydrogen devices also should be investigated. The acceleration of degradation is observed as the catalyst loading decreases, as the reduction of catalyst does not diminish the degradation component but rather amplifies its proportion within the overall catalyst [28, 29]. In PEMFCs, the degradation ratio in the low Pt loading of 0.125 mg_Pt_ cm^−2^ is almost twice as much as the case for the high Pt loading of 0.5 mg_Pt_ cm^−2^ [30]. The ACL performance of PEMWE also decreases significantly at low-Ir loading (e.g., 0.1 mg cm^−2^), while durability losses to this extent were not observed using MEAs with a higher anode loading [31]. Thus, it is imperative to find ways to lower the catalyst loading of PEMFCs and PEMWEs while maintaining high power density and durability.

Overall, the continued commercialization of PEMFCs and PEMWEs has been greatly hindered by the high cost associated with the noble metal catalysts. The optimization of both catalyst materials and electrode structure comprehending oxygen transport mechanisms through the porous structure and critical functions of TPBs in the CLs is necessary to reduce Pt or Ir/IrO_*x*_-based catalyst loadings. In this review, oxygen transport properties in CLs are comprehensively investigated, and the understandings of the PEMFC could guide the development of oxygen transport in PEMWEs. In Sect. 2, the mechanism of oxygen transport in bulk phase and ionomer binders in CCLs in low or ultra-low Pt loading PEMFCs is systematically concluded. In Sect. 3, the strategies for the enhanced oxygen transport focusing on the key materials in CCLs in PEMFCs are highlighted. Sect. 4 offers insights into the existing challenges and development prospects for PEMWEs based on the comprehensive analysis of PEMFCs. Recommendations are proposed to enhance the efficiency of PEMWEs and to further expedite the broad implementation and development of hydrogen infrastructure.

## Oxygen Transport in CCLs in PEMFCs

**Fig. 2:**
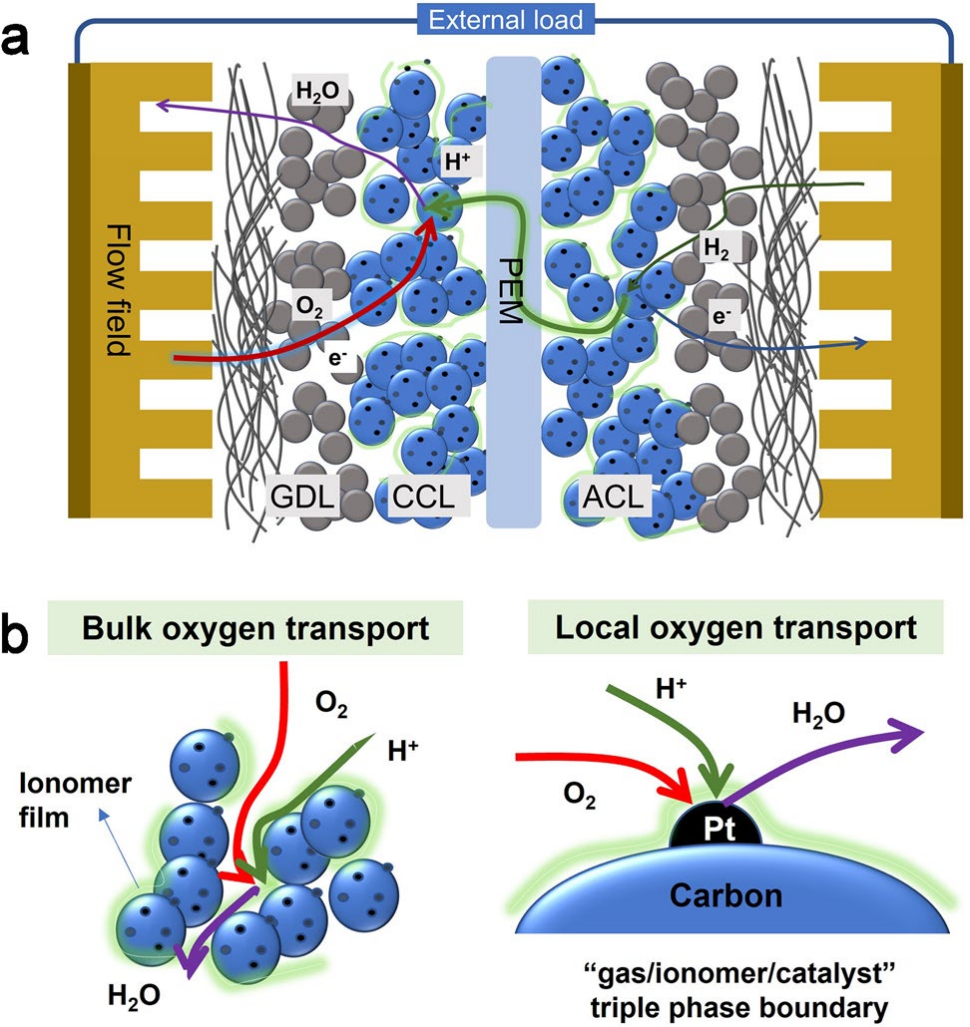
Illustration of PEMFC internal structure.** a** Mass transport, proton, and electron transport process. **b** Bulk and local oxygen transport in CCLs

The structure for PEMFCs depicted in Fig. 2a encompasses the oxygen transport process in the cathode, hydrogen transport in the anode, and the proton transport from active sites in anode to the active site in cathode, passing through the PEM in between, and the electron transport. The mass transport process involving reactants (O_2_ and H_2_) and products (H_2_O) plays a vital role on the fuel cell performance, particularly in relation to concentration polarization at high current densities. It is exacerbated by the reduced Pt loading, leading to the obviously increased oxygen transport resistance in CCLs [22, 23, 32–35]. Oxygen transport in the CCLs is complex including coupling transport process in multi-scale pore structures from nanometer to micron (named bulk transport resistance (*R*_bulk_)) and nano-thickness ionomer films at the “gas/ionomer/catalyst” TPBs (named local oxygen transport resistance (*R*_local_)), as shown in Fig. 2b. Therefore, it is imperative to conduct a more in-depth investigation into the mechanisms of oxygen transport, considering both structural and material perspectives.

### Oxygen Transport Characterization Method in PEMFCs

#### Electrochemical Method

The precise measurement of oxygen transport resistance is indispensable for investigating the mechanism and advancement of oxygen transport issues in PEMFCs. The limiting current method combining the Fick’s law with the Faraday’s law was first utilized by Baker et al. [36] to quantify the total oxygen transport resistance (*R*_total_) in CCLs based on the limiting current (${\text{i}}_{\text{lim}}$), as shown in Eq. (1).

1 $$R_{{{\text{total}}}} = \frac{{c_{{{\text{O}}_{2} }}^{{{\text{Channel}}}} - c_{{{\text{O}}_{2} }}^{{{\text{Pt}}}} }}{{N_{{{\text{O}}_{2} }} }} = nF \cdot \frac{{c_{{{\text{O}}_{2} }}^{{{\text{Channel}}}} }}{{i_{\lim } }}$$

where the parameter *n*, *F*, ${\text{c}}_{{\text{O}}_{2}}^{\text{Channel}},$ and ${\text{c}}_{{\text{O}}_{2}}^{\text{Pt}}$ denote the number of transferred electrons, the Faraday’s constant, the oxygen concentration in the flow field, and Pt surface, respectively.

**Fig. 3:**
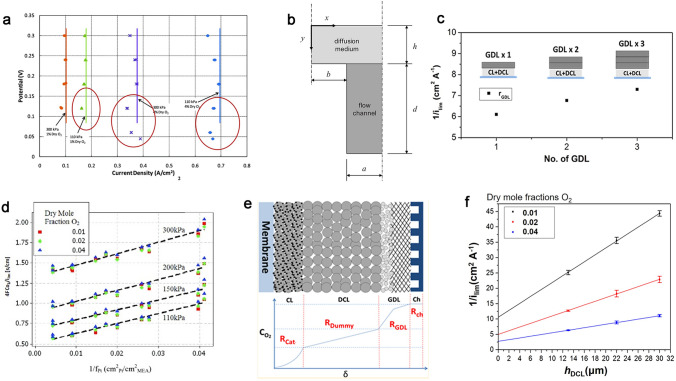
Measurements of oxygen transport resistance in PEMFCs. **a** Typical polarization curves at low oxygen concentrations and low cell potentials. **b** Coordinates in a cross section through the flow field and diffusion medium [37]. Copyright 2009 ECS—The Electrochemical Society. **c** GDL resistance tested varying the number of the GDLs [34]. Copyright 2017 American Chemical Society. **d** *R*_total_ vs. 1/*f*_Pt_, at 0.01, 0.02, and 0.04 dry oxygen mole fractions at 110, 150, 200, and 300 kPa [33]. Copyright 2012 The Electrochemical Society.** e** Illustration of the DCL design with the DCL; **f** 1/*i*_lim_ vs. *h*_DCL_, at 0.01, 0.02, and 0.04 dry oxygen mole fractions [34]. Copyright 2017 American Chemical Society

By conducting a slow scan of the polarization curves, ranging from high voltage to very low voltage at a rate of 5 mV s^−1^, the point of limiting current is reached when there is no further increase in current as the voltage decreases (Fig. 3a). It indicates that the oxygen concentration on the Pt surface ${\text{c}}_{{\text{O}}_{2}}^{\text{Pt}}$ equals zero at this current density [33]. The dry oxygen mole fractions in the cathode inlet gas stream in measurements are always low (e.g., 1%, 4%, 8% O_2_/N_2_) in order to achieve the limiting current, and the operating conditions of low humidity and high gas flow rate are used to avoid the effects of liquid water. It is noted that the current density at low oxygen concentrations and low cell potentials (< 100 mV) firstly decreased and then increased, as shown in red circles in Fig. 3a. The decrease in current is attributed to the occurrence of hydrogen evolution, whereby protons are converted into hydrogen instead of water at the cathode. Moreover, the observed “turn back” increase can be interpreted as a transition from the conventional four-electron process for water production to a two-electron process involving peroxide formation [37]. Thus, the limiting current before the “turn back” at extremely low cell potentials is thus accurately applicable for calculating *R*_local_.

In terms of the complex structure in the cathode of PEMFCs, *R*_total_ could be separated into contributions from channels in flow field (*R*_Ch_), gas diffusion layers (*R*_GDL_), and cathode catalyst layers (*R*_CCL_). *R*_Ch_ could be analyzed accounts for coupled convective diffusion and reactant depletion in Fig. 3b. The experimental formula is given by Baker et al. [36, 37]:

2 $$R_{{{\text{Ch}}}} = A\frac{a}{{D_{{{\text{O}}_{{2}} }}^{{{\text{Ch}}}} }} + B\frac{{2{\text{Ndl}}}}{{Q_{{{\text{dry}}}} }}\left( \frac{273}{T} \right)\frac{{P - P_{{\text{w}}} }}{{P_{{\text{a}}} }}$$

where *A* and *B* are experimental parameters; *a*, *l*, *d*, and *N* are half-width, length, depth, and number of the flow field, respectively; *Q*_dry_ is the total inlet dry gas flow rate at standard temperature and pressure; ${\text{D}}_{{\text{O}}_{2}}^{{\text{C}}{\text{h}}}$ is the diffusion coefficient of oxygen in the flow channel; *P*, *P*_a_, and *P*_w_ are the gas pressure, air pressure, and water vapor partial pressure in flow channel, and *T* is the temperature.

*R*_GDL_ could be measured by *R*_Total_ varying the number of the GDLs (*n*) in different cells under constant compression of the GDL, as shown in Eq. (3) (Fig. 3c) [37]:

3 $$R_{{{\text{GDL}}}} = R_{{{\text{total,}}\;n + 1 {\text{carbon paper}}}} - R_{{{\text{total,}}\;n {\text{carbon paper}}}}$$

Thus, *R*_CCL_ is calculated as Eq. (4):

4 $$R_{{{\text{CCL}}}} = R_{{{\text{total}}}} - R_{{{\text{Ch}}}} - R_{{{\text{GDL}}}}$$

By varying the pressure in PEMFCs, *R*_total_ is separated into a pressure-dependent molecular gas diffusion (*R*_P_) and a pressure-independent component (*R*_NP_) [37, 38]. *R*_NP_ mainly shows the Knudsen oxygen diffusion resistance in CCL in primary pores near the TPBs and through thin ionomer films, while the *R*_P_ is dominated by the contributions of molecular oxygen diffusion resistance in the flow field, the GDL, and large pores in the CCL [39, 40]. If the mole fractions of oxygen, nitrogen, and water vapor were each held constant as *p* changes, *R*_P_ would be a strict multiple of the total gas pressure *p*, and *R*_CCL_ would approach *R*_NP_ in the limit *p* → 0. However, it was more convenient to hold the relative humidity (RH), and hence, the water partial pressure *p*_W_ is constant as *p* varied. Thus, the mole fractions of oxygen, nitrogen, and water vapor are changed. Baker et al. observed that with a constant RH, *R*_CCL_ is still linear in the total pressure with a constant term that consists of more than just *R*_NP_, and they proposed a revised formula based on Eqs. (5) and (6). This method has been widely accepted to depict the oxygen transport in ionomer in low or ultra-low Pt loading CCLs by Ott et al. [41], Lee et al. [42], and others [43, 44], because this method is not limited by the structure of the CCLs.

5 $$R_{{{\text{NP}}}} = {\text{intercept}} - {\text{slope}} \times p_{{\text{w}}} \left( {\frac{{D_{{{\text{ON}}}} }}{{D_{{{\text{Ow}}}} }} - 1} \right) + B\frac{{2{\text{Ndl}}}}{{Q_{{{\text{dry}}}} }}\left( \frac{273}{T} \right)\frac{{p_{{\text{w}}} }}{{p_{{\text{a}}} }}\frac{{D_{{{\text{ON}}}} }}{{D_{{{\text{Ow}}}} }}$$

6 $$R_{{\text{P}}} = R_{{{\text{total}}}} - R_{{{\text{NP}}}}$$

where *D*_ON_ and *D*_OW_ are the binary diffusion coefficients for oxygen relative to nitrogen and water vapor, respectively.

Based on Eqs. (7) and (8), Greszler et al. have made rigorous mathematical derivations and concluded that in the condition that (i) the diluted CCLs possess the same thickness but different Pt loadings and (ii) the oxygen transport resistance in the pores is small enough ($h/\psi < 1.6$), *R*_CCL_ is liner with the roughness factor ${\text{f}}_{\text{Pt}}$, as shown in Fig. 3d [33].

7 $$R_{{{\text{CCL}}}} = \frac{{R_{{{\text{local}}}} }}{{f_{{{\text{Pt}}}} }}(h/\psi )\coth (h/\psi ) = \frac{{R_{{{\text{local}}}} }}{{f_{{{\text{Pt}}}} }}({\text{diluted}}, h/\psi < 1.6 )$$

where *h* denotes the thickness of CL, $\psi = \sqrt {D_{{{\text{O}}_{2} }}^{{{\text{eff}}}} \frac{{hR_{{{\text{Local}}}} }}{{f_{{{\text{Pt}}}} }}}$, ${\text{f}}_{\text{Pt}}=\text{ECSA}{\times L}_{\text{Pt}}$

8 $$R_{{{\text{Total}}}} = \frac{{4Fc_{{{\text{O}}_{{2}} }}^{{{\text{Channel}}}} }}{{i_{{{\text{lim}}}} }} \approx \frac{{R_{{{\text{Local}}}} }}{{f_{{{\text{Pt}}}} }} + R_{{{\text{GDL}}}} + R_{{{\text{MPL}}}}$$

Recently, an experimental approach to measure the oxygen transport resistance in CCLs is proposed with a dual-layer CCL for the first time. Wang et al. [34] added a dummy catalyst layer (DCL) between the real CCLs and the GDLs as shown in Fig. 3e, f, where the DCL has the same structure as the CCL, but the carbon support without Pt particles was used instead of Pt/C catalyst. Therefore, the resistance in the DCL comprises only bulk resistance, as no electrochemical reactions occur within it. In the condition of *h*_DCL_ much higher than ${h}_{\text{CCL}}^{\text{eff}}$, it is reasonable to change the thickness of DCLs to, respectively, quantify the local and bulk resistances via limiting current measurements combined with linear extrapolation, as Eq. (9):

9 $$R_{{{\text{Total}}}} = R_{{{\text{Ch}}}} + R_{{{\text{GDL}}}} + r_{{{\text{bulk}}}} \times h_{{{\text{DCL}}}} + R_{{{\text{local}}}} (h_{{{\text{DCL}}}} \gg h_{{{\text{CCL}}}}^{{{\text{eff}}}} )$$

Moreover, Nonoyama et al. observed that molecular and Knudsen diffusivities only received a 10%-20% change when the temperature goes from 80 to 40 °C, while the oxygen permeation resistance of the ionomer is a much stronger function of temperature, resulting in about a 2.4 times change for the same temperature shift. Thus, the sensitivity difference of transport resistances to operating temperature, including molecular diffusion resistance, Knudsen diffusion resistance, and ionomer permeation resistance, was utilized by to distinguish oxygen transport resistance in CCLs [40].

#### Chemical and Physical Characterization

The pore structure in electrode based on carbon-supported nanoparticles is complex, including the primary pore on carbon surface and the secondary pore constructed by the carbon agglomeration [45]. Primary pores on carbon support are generally tested by nitrogen adsorption methods as it allows assessing the complete range of micro- (pore width < 2 nm), meso- (pore width: 2–50 nm), and to some extend even macropores (pore width > 50 nm). Mercury intrusion porosimetry (MIP) [46] is used for the characterization of the secondary pores on carbon support for larger nanopores and macropores up to 400 μm. The combination of nitrogen adsorption and MIP allows one to obtain pore structure information over a wide range from pore widths < 4 nm up to at least ~ 400 μm, highlighting the importance of these techniques for porous materials characterization. The highly resolution instruments are of great help to extract the shape/size and 3D distribution including connectivity of the material phases and pores. Scanning electron microscope (SEM) uses a narrow focused high-energy electron beam to scan a sample. Through the interaction between the beam and the material, a variety of physical information is generated, which is collected, amplified, and re-imaged to achieve the purpose of the characterization of the material’s microscopic morphology. However, cross-sectional images of the CLs obtained by SEM do not have uniform contrast in the pore area because carbons are often visible behind the cross section, which makes conventional analysis inadequate. Considering the accuracy of SEM images, Ghosh et al. developed a novel method by filling the pores of the CCLs with metalliferous epoxy polymer before using SEM analysis. The uniform contrast across the entire pore area microstructural pore properties was observed without damage the carbon [47, 48]. Based on nanoscale resolution X-ray computed tomography (Nano-CT) combining with statistical information from TEM, X-ray scattering, and Brunauer–Emmett–Teller (BET), the detailed structures of electrode and 3D agglomerates of solid carbon could be observed as spheres inscribed to segmented images [49]. From the above image analysis, three-dimensional reconstruction could be realized and facilitated the further study. The segmented images of CLs showing the electrode structures could also be investigated by focused ion beam–scanning electron microscope (FIB–SEM) integrated by the tomography analysis, which allows a complete consideration of the electrode morphology, providing several key pore-structural information, e.g., porosity, diffusivity, and tortuosity [50]. With the help of the above accurate pore morphologies, the numerical simulation algorithms, three-dimensional reconstruction, or even machine learning could be conducted and provide more information within the CLs for the study of relationship between microstructure and mass transport [51].

The morphology and microstructure of ionomer thin films coated on the catalyst and carbon supports are also crucial, yet there is a scarcity of experimental data available for their characterization due to the nanoscale nature of ionomer within CCLs. The primary challenge in distinguishing between PFSA-based ionomer and carbon lies in the destruction of ionomer thin films due to electron irradiation with the high required beam sensitive and high rate of radiolysis for sufficient signal-to-noise ratios. Based on electron tomography performed in a high-angle annular dark-field scanning transmission electron microscope (HAADF-STEM), Lopez-Haro reported the three-dimensional morphology of the Nafion thin films surrounding the carbon particles after Cs^+^ [52]. The higher atomic number compared to carbon enhances the contrast and provides a more precise and distinct distribution of ionomer films. This method is similarly employed by Sun et al. [53], yielding distinct nano- and sub-nanostructures at the interfaces comprising ionomers and Pt. However, this method does not modify the structure of the PFSA-based ionomers, nor does it address the issue of electron irradiation caused by beam sensitivity. The efficient electron imaging method of the PFSA-based ionomer can be conducted with cryo-electron tomography (Cryo-ET), which significantly mitigates the rate of radiolysis due to the vitrification by rapid freezing [54, 55]. This method could integrate three-dimensional molecular-level imaging with the highest level of structural preservation that can be physically attained [56, 57].

On the side, quantitative investigation of the ionomer and the catalyst/carbon support can be observed indirectly through the material modulus, sensitive and conductive [58–60]. For example, due to that the modulus of the ionomer is much lower and the adhesion is much higher than that of the catalyst/carbon supports, Cheng et al. and Su et al. successfully decoupled the ionomer distribution and ionomer films thickness in the CCLs based on AFM [59, 60]. The characterization of the adsorption process of the ionomer thin films on the catalyst/carbon support always depends on in situ or semi-situ experiments. Electrochemical quartz-crystal microbalance (EQCM) with high mass resolution enables determination of the tiny mass changes of ionomer adsorption during electrochemical reactions. Li et al. analyzed the ionomer adsorption on carbon supports during the degradation process of CCLs with the utilization of EQCM and found that the surface oxygen-containing functional groups make an influence on the adsorption of ionomer films and further affect the ionomer distribution in CCLs [61]. Sarah et al. investigated the detail ionomer adsorption using the QCM to model surfaces charge densities under a variety of solvent environments and probed the origin of the complexities of ionomer/particle adsorption interaction in inks [62]. Through the combination of in situ AFM and EQCM, the positive adsorption process of ionomer can be characterized more carefully [63]. The deeper adsorption characteristics of the ionomer main chain and side chain structure on the catalyst/carbon support at the TPBs can be explored through the infrared reflection absorption spectroscopy (IRRAS) [64], in situ attenuated total reflection fourier-transform infrared (ATR-FTIR) spectroscopy [65], and surface enhanced infrared absorption spectroscopy (SEIRAS) [66] through detecting of molecular chemical bonds.

The aforementioned methods, which involve conducting experiments in electrolyte solutions or unique MEA configurations, offer a comprehensive correlation between the ionomer and substrate. The microstructure properties of thickness-dependent ionomer films, such as nanophase separation and water uptake, are necessary to verified. Grazing-incidence small-angle X-ray scattering (GISAXS) patterns are employed for the nanophase separation in ultrathin ionomer films, and small-angle X-ray scattering (SAXS) is used for the crystalline structures of the bulk ionomer membranes [67]. Further, small-angle neutron scattering (SANS) and 19F NMR are commonly used for the investigation of ionomer morphology and structure in catalyst inks [68]. These advanced characterization methods promote the study of local oxygen transport mechanism.

### Mechanism of Oxygen Transport in Pore Structure

**Fig. 4:**
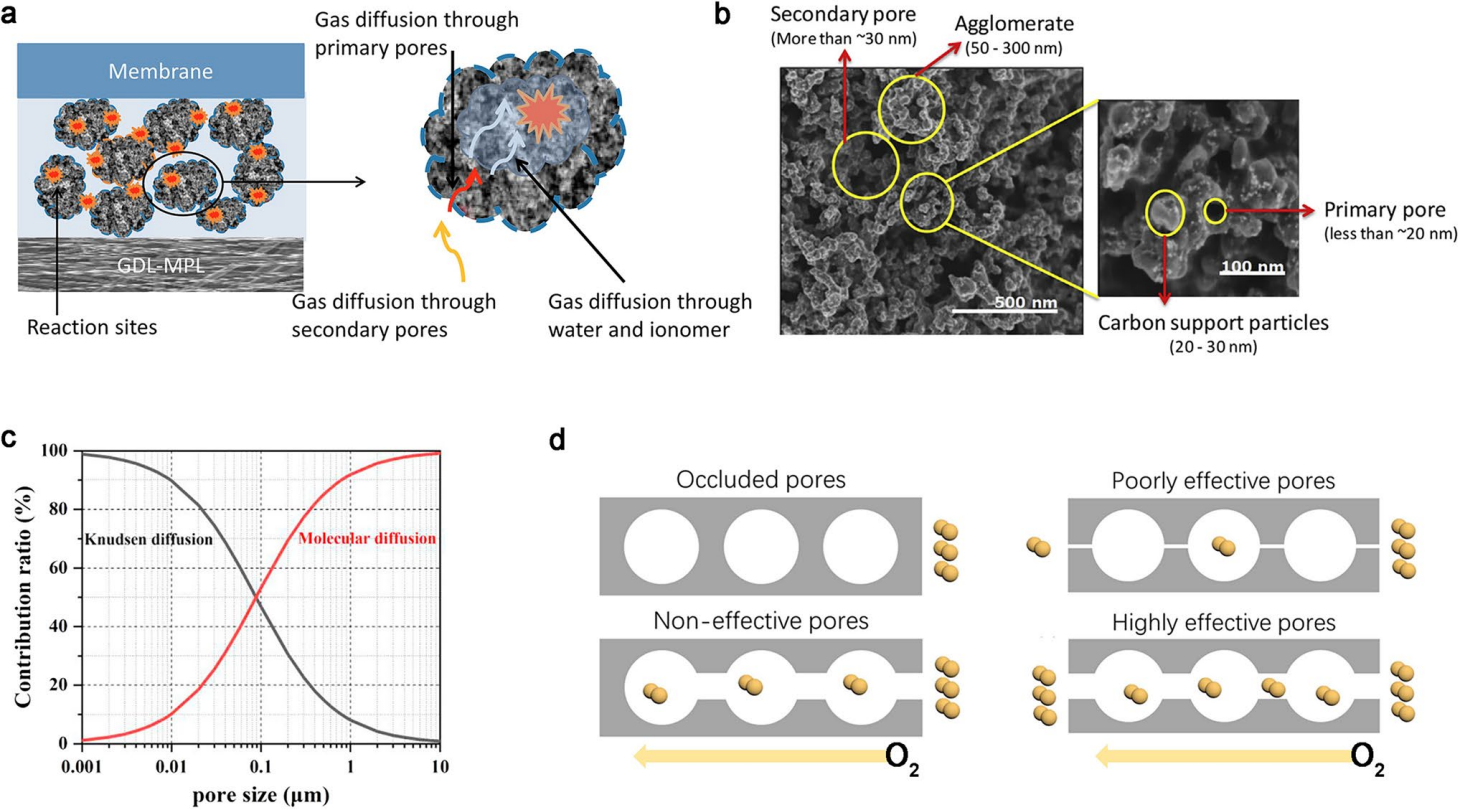
Bulk oxygen transport in pore structure in CCLs.** a** Oxygen transport illustration in primary and secondary pores [45]. Copyright 2019 Elsevier B.V., and **b** SEM images for the primary and secondary pores [69]. Copyright 2019 Hydrogen Energy Publications LLC. Published by Elsevier Ltd. **c** Contribution ratio between pore size and oxygen diffusion [70]. Copyright 2020 Chinese Materials Research Society. **d** Four pore types of occluded, non-effective, poorly effective, and highly effective pores [71]. Copyright 2019 The Electrochemical Society

Detailed insights into the pore structure are essential as it controls the oxygen transport phenomena in the pores as shown in Fig. 4a. Salari et al. combined the ex situ and the in situ gas diffusion measurements to investigate the contributions to oxygen transport resistance between primary pores and the secondary pores (Fig. 4b). They found that the *R*_bulk_ is almost caused by secondary pores, while the primary pores’ direct contribution to the diffusion resistance is negligible [45]. However, the size of the primary pores has a notable impact on the ionomer–water relative diffusivity, which is related to *R*_local_ and will be discussed in detail in the next section. In order to arrive at a comprehensive structure-relationship characterization of such complex CCLs, a combination of complimentary experimental techniques is necessary. The key parameters for characterizing the electrode microstructure of the CCLs are the pore size distribution, porosity (*ɛ*), and tortuosity (*τ*).

The pore size combined with the mean free path of oxygen could be the basis to divide to the oxygen diffusion into the molecular diffusion and the Knudsen diffusion [39, 72], as shown in Fig. 4c. If the pore size significantly exceeds the mean free path, molecular diffusion prevails, and the resistance to diffusion arises from intermolecular collisions. Conversely, Knudsen diffusion dominates, and the resistance to diffusion results from gas molecule collisions with pore walls. The oxygen effective diffusion coefficient ${\text{D}}_{{\text{O}}_{2}}^{\text{eff}}$ based on molecular diffusion is significantly higher, by three orders of magnitude higher than that in the Knudsen diffusion [73]. It is clearly demonstrated that increasing the pore size is advantageous for facilitating oxygen transport [70].

The analysis of pore size distribution alone, without considering porosity and tortuosity, is insufficient to fully capture the oxygen transport phenomenon. Porosity is directly linked to pore volume, while tortuosity quantifies the elongation of transport paths caused by the porous structure compared to a straight line. The relationship between porosity *ɛ* and oxygen effective diffusion coefficient ${\text{D}}_{{\text{O}}_{2}}^{\text{eff}}$ is usually depicted by the Bruggeman approximation, especially in mathematic simulations as shown in Eq. (10) [74]. And the Bruggeman relation of tortuosity τ and porosity *ɛ* is shown in Eq. (11):

10 $$D_{{{\text{O}}_{{2}} }}^{{{\text{eff}}}} = D_{{{\text{o}}_{{2}} }} \cdot \varepsilon^{1 + n/n}$$

11 $$\tau = \varepsilon^{ - \alpha }$$

where ${\text{D}}_{{\text{o}}_{2}}$ is oxygen diffusivity in free space, *n* equals 2, and *α* equals 0.5 for classical spherical particles.

However, the predicted tortuosity and porosity value based on the Bruggeman approximation are usually far away from the experimentally obtained value, illustrating that the classical Bruggeman approximation for spherical particles can lead to large errors. Landesfeind and Gasteiger et al. have demonstrated that the Bruggeman estimation for spherical particles largely underestimates the oxygen transport resistances through the porous separators [75]. To further investigate this huge gap, Cheng et al. accurately quantified ${\text{D}}_{{\text{O}}_{2}}^{\text{eff}}$ in the porous electrode based on the dual-layer CCLs design. They surprisingly found a much smaller porosity calculated by Eqs. (10–11) than MIP results, which is suggested to result from the occluded pores (pores not connected to the main void space) that do not contribute to the oxygen transport as illustrated in Fig. 4d, and only interconnected pores are taken into accounts [71].

Further, the thickness of CCLs could be another factor to impact *R*_bulk_ because it determines the transport path of oxygen in the pore. It is found by Sassin et al. that the pore size distribution remains unchanged as thickness of CCLs increases from 3.8 to 11.8 mm, but porosity decreases with further increasing CL thickness [76]. The design of a porous and stable structure for the CCLs is crucial in order to enhance oxygen transport within the porous electrode and thus increases the oxygen concentration at the TPBs of both catalyst and ionomer. This can be achieved through efficient methods, such as pore formation [77]. It is noting that with the reducing of Pt loading, the thickness of CCLs decreased and the bulk oxygen transport resistance is alleviated and thus the *R*_local_ oxygen transport resistance is gradually prominent.

### Mechanism of Oxygen Transport at TPBs

**Fig. 5:**
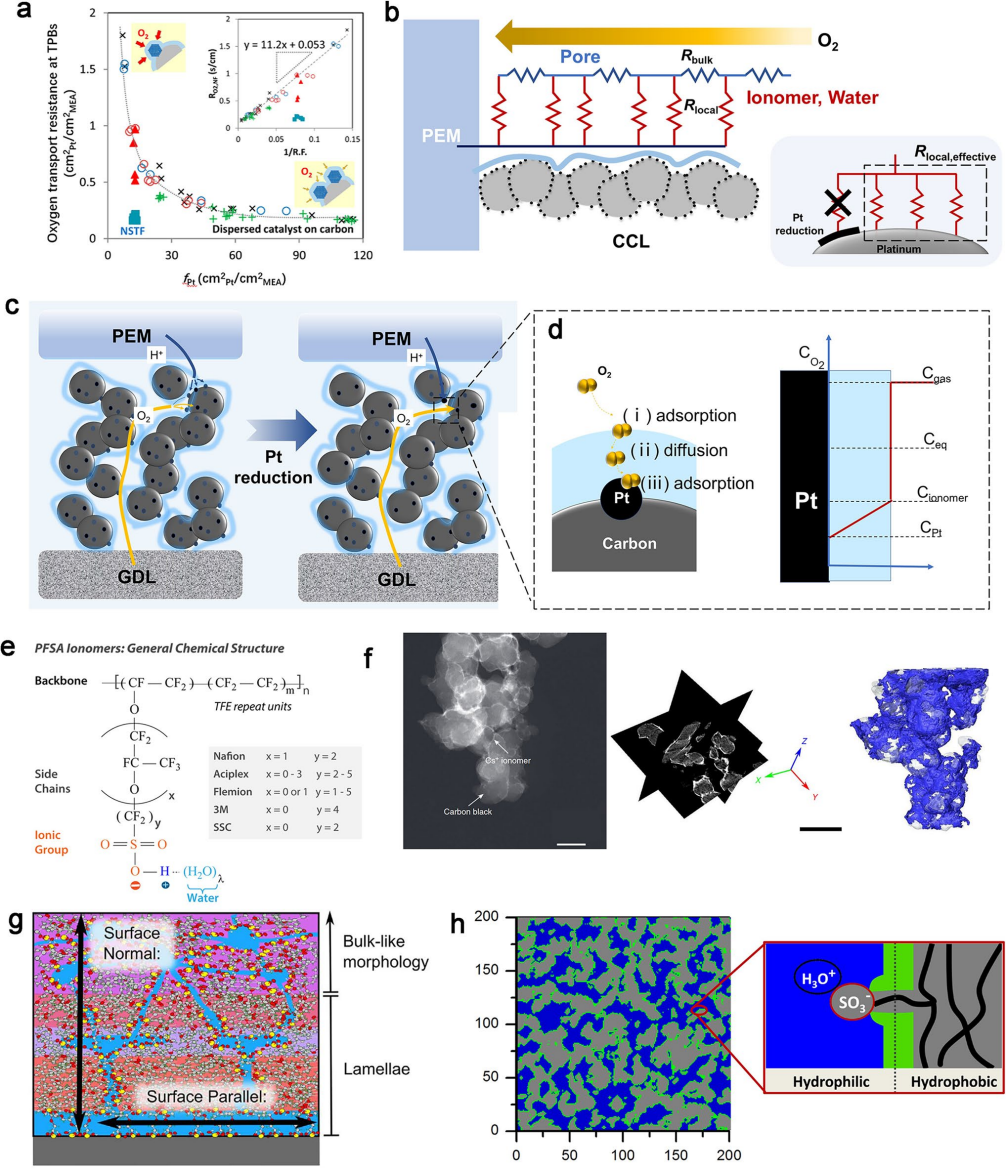
Mechanism of *R*_local_ in CCLs in PEMFCs.** a** Oxygen transport resistance at TPBs as a function of the Pt roughness factor *f*_Pt_ [23]. Copyright 2016 American Chemical Society. **b** Schematics of macro-homogeneous model with reduced Pt loading; and illustration of oxygen transport process based on reduced Pt loading [35]. Copyright 2013 The Electrochemical Society. **c** Illustration of oxygen transport path in CCLs with the reduced Pt loading. **d** Adsorption-solution model for oxygen transport at TPBs. **e** Chemical structure of the PFSA ionomer[78]. Copyright 2017 American Chemical Society. **f** 2D imaging of the Nafion/CB nanocomposites and its electron tomography imaging [52]. Copyright 2025 Springer Nature Limited. **g** Schematic of Nafion thin-film structure [79]. Copyright 2018 Elsevier Ltd. **h** Modulus of Nafion as a function of film thickness [80]. Copyright 2014 American Chemical Society

The local oxygen transport process at the TPBs has been found as the main oxygen transport resistance in low or ultra-low Pt loading electrode. It has been observed to exhibit a sharp increase with decreasing Pt loadings, as depicted in Fig. 5a [23], and the further detail calculation by Wang et al. also proved that the *R*_local_ accounts for 77% of the *R*_total_ in CCLs as the Pt loading reduced to 0.05 mg_Pt_ cm^−2^ [34].

As shown in Eq. (12), the *R*_local_ here refers to the local oxygen transport resistance normalized to electrochemical surface area (ECSA) and* r*_local_ refers to the local oxygen transport resistance normalized to Pt surface area. The *R*_local_ showed a strong dependency on ECSA and Pt loading *L*_Pt_, also known as the Pt roughness factor *f*_Pt_ [23].

12 $$R_{{{\text{local}}}} = \frac{{r_{{{\text{local}}}} }}{{f_{{{\text{Pt}}}} }}$$

13 $$f_{{{\text{Pt}}}} = {\text{ECSA}} \times L_{{{\text{Pt}}}}$$

Ono et al. have brought about the macro-homogeneous model (Fig. 5b) to divide the *R*_bulk_ in thickness direction of CCLs, and the *R*_local_ in direction toward to Pt surface [35, 81]. The Pt roughness factor *f*_Pt_ is the dominant factor at the high current density because more oxygen must be delivered to a smaller active surface as the Pt loading decreased, resulting in a higher apparent electrode oxygen transport resistance, as depicted in Fig. 5c [23]. Greszler et al. observed that the reduced Pt loading makes the partial pressure of oxygen decrease at the Pt surface significantly [33], thereby impeding the oxygen transport process on the Pt surface. The ECSA is a significant parameter used to represent the intrinsic activity of the Pt catalysts with proton transport paths network, which could be calculated by using the eversible adsorption/desorption area of certain species such as Cu, CO, and H. It would be deeply influenced by catalyst morphology. For example, the ECSA could be increased as the Pt utilization increased due to uniform dispersion or reduced particle size [82].

With the constant ECSA and Pt loading, the primary challenge of the local oxygen transport at TPBs is *r*_local_. It depends on the oxygen transport properties in the thin ionomer films coating on the Pt surface, which is of tremendous importance for the proton conduction and construction of CLs, while it is existed as a barrier for gas transport and results in the concentration polarization. The transport of oxygen through thin ionomer films has been widely considered to follow a solution-diffusion mechanism, where oxygen molecules firstly adsorb on the gas/ionomer interface, then transport in ionomer film, and finally adsorb at the ionomer/Pt interface (Fig. 5d).

This process depends strongly on the structure of the thin ionomer films [83]. The PFSA ionomer in CCLs uniquely consists of the hydrophilic sulfonic groups and the hydrophobic PTFE backbones as shown in Fig. 5e, which shows phase-separated structure providing fantastic proton conductions. Although the chemical composition is similar between the PEM and ionomer films coated on carbon support and catalyst nanoparticles, the thickness plays an important role on the physical and chemical properties, which is closely linked to the oxygen and proton transport. In order to high-resolution technologies, the studies of thickness of ionomer film in CCLs have been put into effect. Lopez-Haro et al. observed the ionomer ultrathin films covered on carbon surface based on a representative HAADF-STEM image of Cs^+^ stained, and the thickness of ionomer is around 7 nm as shown in Fig. 5f. The increasing ionomer content is responsible for the ionomer coverage on the carbon support rather than the thickness, until the ionomer is too much in CCLs [52]. The nanoscale properties of PFSA ionomers that the thickness approaches the characteristic domain size of the ionomers result in confinement effects and substrate effects coated on the carbon support and catalyst in CCLs, whose structure can drastically differ from the bulk membranes between cathode and anode. Weber et al. attributed the significant oxygen transport loss in the ionomer in CCLs to the confinement effect and the substrate effect due to the nano-thickness properties, which will reduce the water uptake and thus influence the transport behaviors [22, 58].

The thickness-dependent ionic-domain structure is well investigated in terms of the self-assembled method of ionomer thin films on a silicon substrate. The confinement effect and the interaction with the substrate could result the changes in arrangement of the side chain and the backbone, which will further make the surface changes from hydrophobic to hydrophilic [84]. The thickness dependence of ionomer properties investigated based on the in situ neutron reflectometry suggests that as the film thickness below 12 nm, the entire film consists of lamellae, while the thickness between 12 and 42 nm, and a non-lamellar bulk-like layer forms between the lamellae and vapor. Further, as the thickness is above 60 nm, the bulk-like layer thickness exceeds the radius of gyration for thin-film Nafion [79], as shown in Fig. 5g. Compared to the thicker membranes, the nanoscale films exhibit lower water uptake, which concluded in the sample varies non-monotonically with thickness, and can be ordered as: thin-film < truncated < thick-film [79, 80, 85, 86]. Thus, the structure of thin ionomer films could be controlled by processing-induced orientation as well as the thickness-dependent contribution of polymer domains.

Considering the aforementioned confinement and the influence of substrate on ionomer, the distinctive structure of ionomer films governs the pathway for oxygen diffusion in ionomer. The ionomer films consisting of hydrophilic and hydrophobic domains, free volumes as well as the intermediate phase between the hydrophilic and hydrophobic phases are shown in Fig. 5h. It was observed that oxygen primarily undergoes transportation within the hydrophilic region due to the dense structure of PTFE main chains, and it has been demonstrated that the permeability of oxygen in water is four times higher than that in PTFE [87]. An alternative perspective arises from molecular dynamics (MD) simulation, which posits that oxygen is transported across the volumes between hydrophilic and hydrophobic interface. It is widely considered the hydrophilic and hydrophobic phase separation structure serves as a crucial foundation for facilitating oxygen transport although the exact transport route of oxygen in ionomer films needs further investigation. In addition, the thickness of ionomer films is a factor for the length of oxygen transport pathway. With the ionomer/carbon (I/C) ratio increased, the ionomer films thickness increased, and the ionomer agglomeration occurred, both of which could increase the oxygen transport resistance in ionomer [88].

**Fig. 6:**
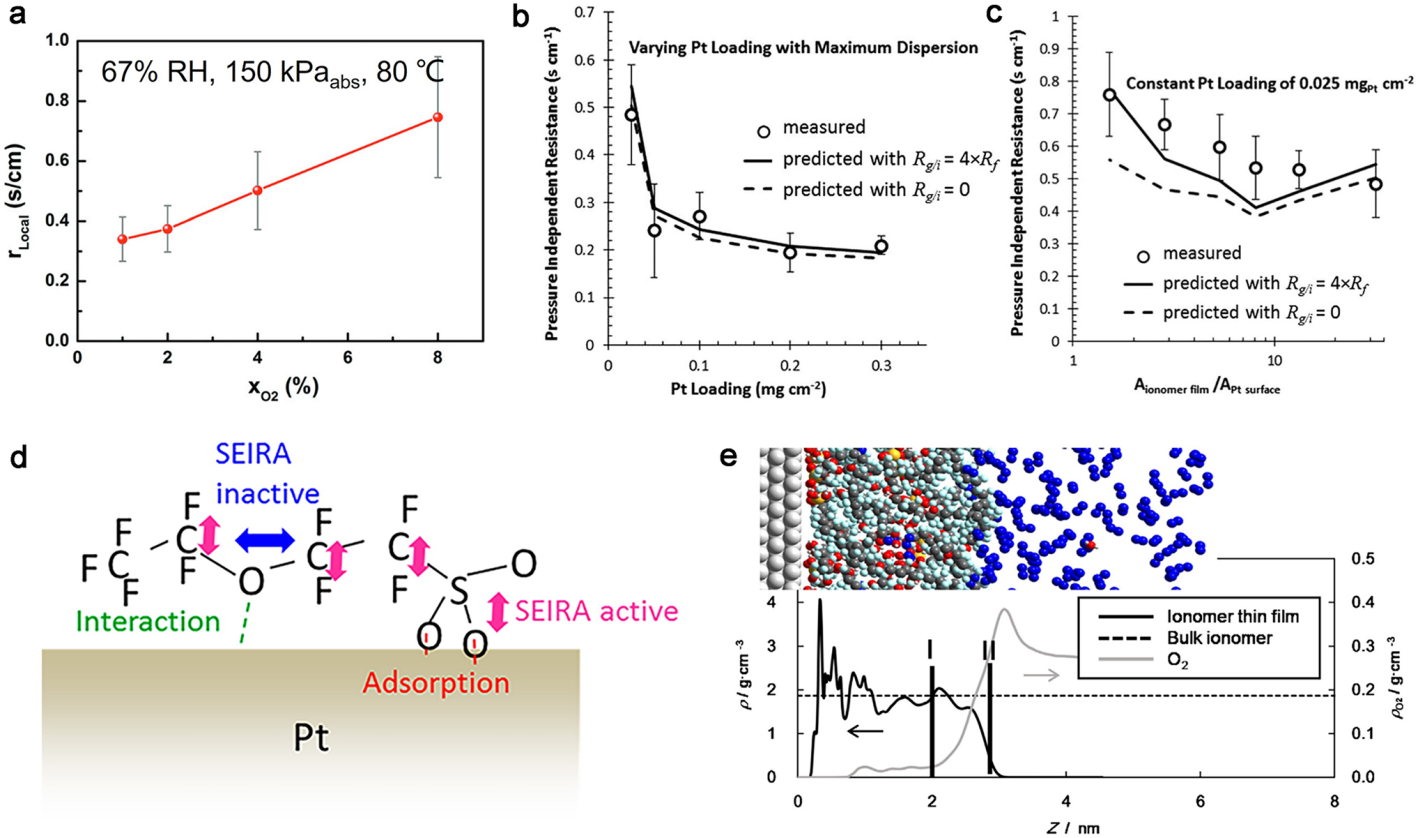
Mechanism of *R*_local_ at the gas/ionomer and ionomer/Pt interface. **a** *r*_local_ at 0.01, 0.02, and 0.04 dry oxygen mole fractions [87]. Copyright the Owner Societies 2017. **b** *R*_NF_ vs. Pt loadings with varying Pt loading with constant dispersions; **c** *R*_NF_ vs. A_ionomer films_/A_Pt surface_ with constant Pt loading of 0.025 mg_Pt_ cm^−2^ with varying Pt dispersions [83]. Copyright 2013 The Electrochemical Society. **d** Illustration of adsorbed structure of -SO_3_^−^ on Pt surface [92]. Copyright 2017 American Chemical Society. **e** A snapshot of the ionomer/Pt interface and density profiles of the ionomer (shown as solid black line) and oxygen molecules (shown as solid gray line) [93]. Copyright 2015 Elsevier Ltd

When considering the nanoscales of ionomer films, the adsorption at the gas/ionomer and ionomer/Pt interface is equally significant in comparison with the diffusion within the ionomer. Oxygen reaches the gas/ionomer interface and adsorbs, which determines the speed of subsequent diffusion in ionomer films and is usually negligible in the conventional thick PEMs. The abnormal gas/ionomer interface resistance in operando conditions was assessed by Shen et al., and they found that *R*_local_ in ionomer was increased by the oxygen concentration, as shown in Fig. 6a. It is suggested by the solution–dissolution model that the oxygen adsorption on the gas/ionomer interface does not obey Henry’s law, because the oxygen adsorption would reach a saturated station due to the few adsorption sites [87]. Based on the sensitive QCM, Li et al. proved that the oxygen adsorption on the thin ionomer films could be fitting with the Toth adsorption model, one of the near logarithmic adsorption, as a consequence of the uneven adsorption properties of thin ionomer films due to separation of hydrophilic and hydrophobic phases [89]. This insufficient adsorption capacity of oxygen could result in a high *R*_local_, and the adsorption of oxygen on the gas/ionomer interface cannot be ignored, especially in the low or ultra-low Pt loading (0.025 mg_Pt_ cm^−2^) as shown in Fig. 6b, c [83]. However, the measurements of oxygen adsorption process in fuel cells are scarce as a consequence of the nanoscale confinement. Kudo and Suzuki et al. developed an electrochemical cell to separate internal and external oxygen transport resistance of the ionomer thin films. Pt electrodes are printed on a quartz plate, and ionomer films were spin-coated on the electrodes, while oxygen was diluted with nitrogen and supplied to the cell. The ex situ interface resistance could be quantified by limiting current measurements, and it is concluded that the dissolution of oxygen on the gas/ionomer interface rather than the oxygen diffusion in the bulk ionomer is the main reason for the observed *R*_local_ [90, 91].

On the other hand, the ionomer/Pt interface is the place that ORR occurred. The strong adsorption of the -SO_3_^−^ groups of ionomer toward Pt catalyst will not only poison the catalyst [92], but also form hydration-dependent multilamellar nanostructure at the Pt interface (Fig. 6d). Ionomer could form an interface adjacent to a Pt substrate, where becomes hydrophilic when in contact with the catalyst surface, causing a long-range restructuring of its chains and changes ionomer structure, and transport therein [78]. Studies by MD simulations observed that the density layer on the surface of ionomer and Pt makes the oxygen transport more restrictive and hinders the oxygen transport, as shown in Fig. 6e [93, 94]. The oxygen transport behavior at this interface requires further experimentation to obtain additional evidence.

### Influencing Factor of Oxygen Transport in CCLs

#### Effect of CCL Fabrication on Oxygen Transport

**Fig. 7:**
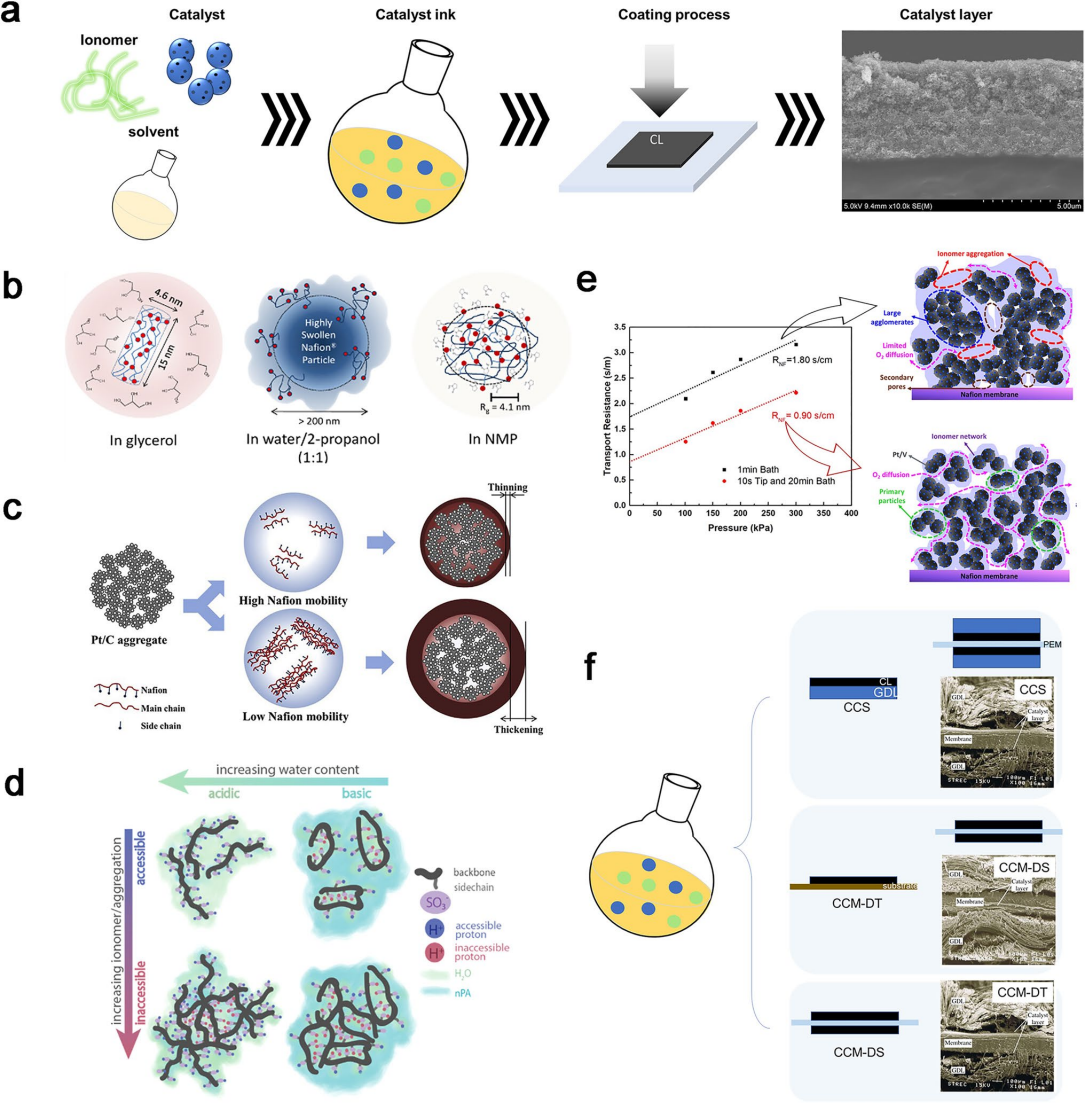
CCL fabrication methods. **a** Illustration for the CCL preparation. **b** Three types of particle morphology in glycerol, water/isopropanol mixtures, N-methylpyrrolidone [68]. Copyright 2012 American Chemical Society. **c** Schematic of the effect of the ionomer mobility on the Pt/C-Nafion agglomerate structure [97]. Copyright 2016 Hydrogen Energy Publications LLC. **d** Schematic of 2D slice of potential structure representing individual chains and aggregates [98]. Copyright 2018 American Chemical Society. **e** Oxygen transport resistance with catalyst inks processed by 1 min bath and 10 s tip and 20 min bath sonication [99]. Copyright 2019 American Chemical Society. **f** Fabrication illustration and SEM images for the CCS and CCM coating methods [100, 101]. Copyright 2020 The Chemical Industry and Engineering Society of China, and Chemical Industry Press Co., Ltd. Copyright 2010 Elsevier Ltd

The catalyst ink for PEMFCs typically comprises carbon-supported catalysts, ionomer and solvents, all of which need to be precisely adjusted to attain the desired ink properties necessary for forming a uniform and continuous CL. The fabrication process, which consists of the dispersion of catalysts inks and different coating processes as illustrated in Fig. 7a, is a primary factor influencing the structural characteristics. The morphology and aggregation size of ionomer in the inks play a key role in the ionomer distribution, since the ionomer/carbon interface in CCLs is formed by self-assembly between ionomer and carbon support/catalyst nanoparticles. There exists micro- and nanoscale interactions including ionomer/carbon-supported nanoparticle, carbon-supported nanoparticle/solvent, and ionomer/solvent. This intricate multiscale system can affect microstructures, rheology, and stability of the ink [95], which could further influence the size of carbon aggregation and distribution of the ionomer in CCLs [96].

The solvent serves as vehicles of the carbon-supported catalysts and ionomer, widely using water, organic solvents, such as ethanol, isopropanol (IPA), N-propanol (NPA), N-methylpyrrolidone (NMP), glycerol, formamide, and N-methyl formamide (NMF), or a mixture of water and solvents. The properties of the solvent relating to its ability to separate opposite charges in solution generally quantified by the static dielectric constant (*Ɛ*), which is also reflects the polarity of the solvent and affect the formation of catalyst/ionomer interfaces and the size of carbon agglomeration. The ionomer with its distinct hydrophilic and hydrophobic structure forms a miscible solution phase at dielectric constants greater than 10, a micellar colloidal phase for 10 > *Ɛ* > 3, and precipitates when *Ɛ* < 3 [102, 103]. The SANS modeling conducted by Welch reveals three distinct particle morphologies in solvents with different *Ɛ*, as shown in Fig. 7b [68]. In addition, the mobility of ionomer films is affected by various solvents with different viscosities, thereby resulting in diverse morphologies of the ionomer agglomerates and ultimately impacting the CL structures as illustrated in Fig. 7c [97, 104]. The water content is also pivotal for the ionomer aggregations in catalyst inks, due to the interaction between water and –SO_3_^−^ by the electrostatic interactions. The addition of water changes the dielectric constant and polarity, and the electrostatic repulsion is enhanced, which can expose the side chain of ionomer and generate smaller ionomer agglomerations, as shown in Fig. 7d [98].

Ball-milling process is usually used to break up aggregations in the solvent to form a uniform and stable ink dispersion for further coating process [105]. A combination of brief tip sonication followed by bath sonication also shows potential at breaking up agglomerates, and the ultrasonic dispersing time could impact structure and oxygen transport resistances (Fig. 7e) [99]. The insufficient dispersion will cause large catalyst agglomerates in the inks and convert to the CCL structure, leading to higher oxygen transport resistance than properly dispersed catalyst, while the excessive dispersion will cause the catalyst to fall off the carbon supports and reduce ECSA. The morphology of the ionomer aggregations in the ionomer dispersions could translate a difference in the ionomer distribution and various pore structures in the CLs. Gisu et al. suggest that for the small ionomer aggregations, Pt nanoparticles are barely exposed to the surface because of a homogeneous ionomer coverage on the catalysts, while an isolated bulky ionomer phase was observed for the large ionomer agglomerations [106]. The mechanism of aggregations formation involving ionomers and carbon-supported catalysts has been investigated; however, the impact of ink on the pore structure and the distribution of ionomers within the catalyst-coated layer (CCL) require further study and exploration.

The coating process of CCLs could be divided into catalyst-coated substrate (CCS) and catalyst-coated membrane (CCM), while the CCM can be characterized for catalyst-coated membrane by direct spray (CCM-DS) and decal transfer (CCM-DT), as shown in Fig. 7f. Studies have demonstrated that CCM fabrication methods exhibit superior performance compared to CCS, primarily attributed to enhanced catalyst utilization and improved oxygen transport processes [100]. The CCS method always conclude a hot-pressing with a high temperature and pressure after coating the catalyst on the GDLs, in order to make a uniform contact between PEM and CLs. Thus, it will reduce the thickness of the CCLs and the pore volume of the GDLs and CLs. The CCLs prepared by CCM are more uniform and have less damage to the pore structure. In the CCS method, catalyst nanoparticles will penetrate the pore structure of GDL, and it reduced the utilization rate of Pt and resulting in uneven catalyst distribution.

#### Effect of Key Materials on Oxygen Transport

The effects of key materials in CCLs, including electrocatalyst, carbon support, and ionomer, on the oxygen transport are worth paying attention to in terms of the strong interaction between interfaces and pore structure of the electrode. This interaction between key materials properties and oxygen transport process is enlarged especially during the degradation process in the long time and complex operating conditions.

##### Electrocatalyst

**Fig. 8:**
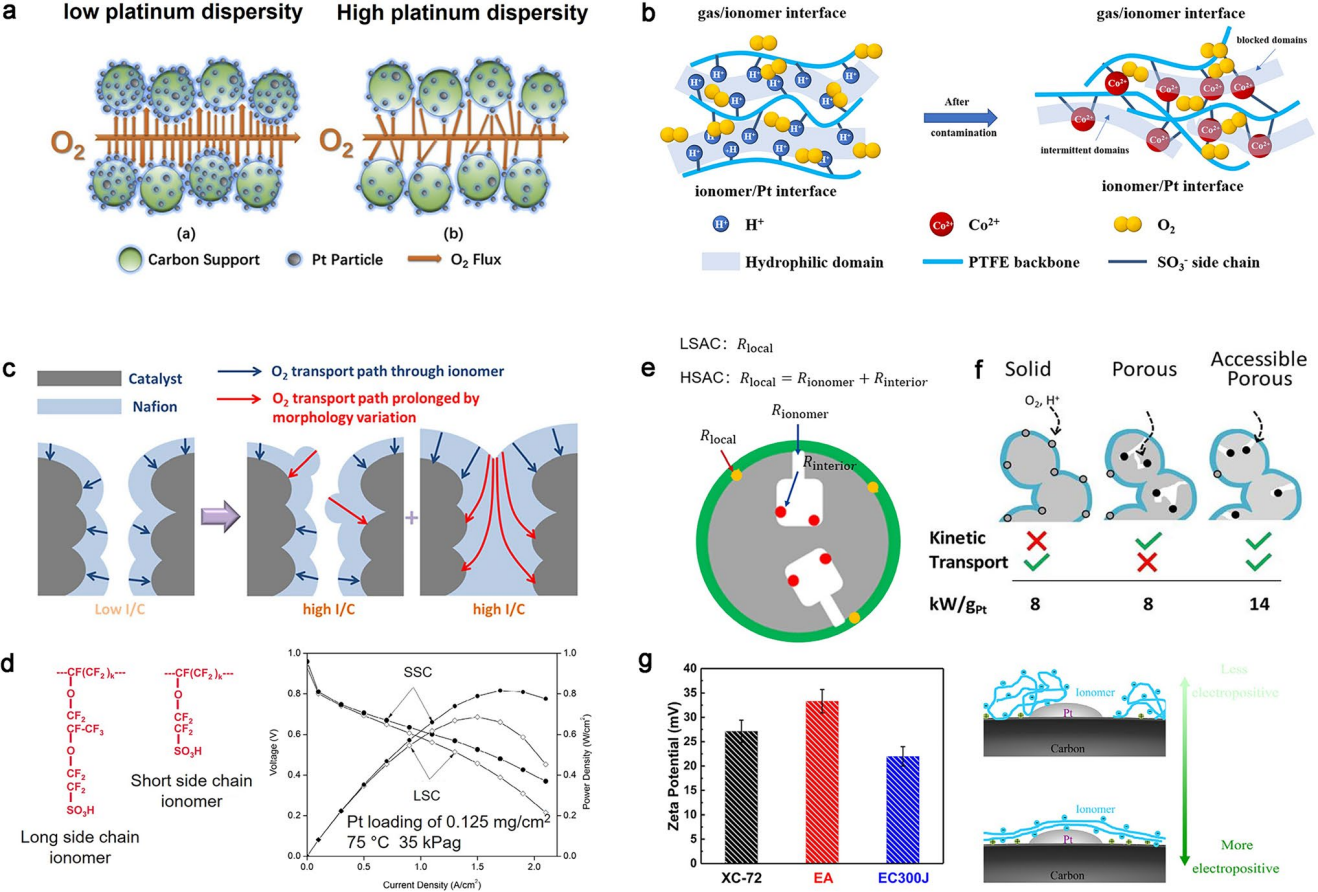
Effects of key materials on oxygen transport resistance.** a** Oxygen transport process with low and high Pt dispersity [112]. Copyright 2019 Elsevier Ltd. **b** Oxygen transport mechanism in original and Co^2+^ contaminated ionomer film [113]. Copyright 2022 Elsevier B.V. **c** Local transport process with increased ionomer content [88]. Copyright 2019 The Electrochemical Society. **d** Polymer structure for long-side chain and short-side chain ionomer (left) [114], Copyright 2011 Elsevier B.V., and their performance (right) [30]. Copyright 2018 Elsevier Ltd. **e** Local oxygen transport mechanism of LSAC and HSAC [115]. Open access.** f** ORR kinetic and transport (oxygen and proton) characteristics of CCLs made from three types of carbon [116]. Copyright 2018 American Chemical Society. **g** Zeta potentials of the three types of carbon supports and sketch of the hypothesized ionomer distribution on the different carbon supports [117]. Copyright 2020 Elsevier Ltd

The size and distribution of electrocatalyst particles on the carbon support are associated with the construction of TPBs. It primarily impacts the ECSA and, in turn, affects local oxygen transport according to Eqs. (12) and (13) [107–109]. Gummalla et al. compared the initial performance and decay trends of electrodes with Pt_3_Co catalysts of three mean particle sizes (4.9, 8.1, and 14.8 nm) with identical Pt loadings and found that the electrode with smallest particle size shows the highest ECSA, which is believed to benefit the local oxygen transport process, but the smallest particle size results in the fast degradation causing by the severe particle agglomerations [110, 111]. As the oxygen transport path illustrated in Fig. 8a, it is suggested that the more uniform Pt distribution could give oxygen more routes to the reaction site and overcome performance loss under high current density [112].

On the side, Pt nanoparticles experience dissolution [118–120], Oswald ripening, detachment, and coalescence [121] in the long-term operation, which are not only decrease the intrinsic ORR activity, but also responsible for the decreased ECSA and Pt loading and increased *R*_local_, ultimately leading to a further decline in fuel cell performance. The cation leaching decay process of Pt-Alloy (Fe, Co, Ni, etc.) catalysts leads to addition oxygen transport resistance. In terms of Eqs. (12) and (13), oxygen transport ability in ionomer depicted by *r*_local_ is another factor influencing *R*_local_ except for the ECSA and Pt loading. It depends deeply on the ionomer morphology and distribution. The Pt-Alloy catalyst suffers the alloy cation leaching, which would not only decrease ECSA, but also destroy the ionomer phase separation structure at the TPBs and decrease *R*_local_ indirectly [110, 113, 122]. Li et al. found that the Co^2+^ could increase the modules and decrease water content of ionomer thin films due to the high affinity between Co^2+^ and –SO_3_^−^. It could make the ionomer chain cross-linking and destroy the hydrophilic and hydrophobic domains, which increases the oxygen transport resistance in ionomer as shown in Fig. 8b [113]. The oxygen transport properties for the fancy catalysts such as core–shell structured catalysts with multilayer or monolayer platinum shell and various kinds of cores still need further study.

##### PFSA *Ionomer*

The crucial role of ionomer in CCLs has been emphasized above, encompassing its functions as a binder and pathway for proton transport, along with its impact on local and bulk transport properties. Ionomer content has been considered to affect not only the thin ionomer films coverage and thickness, but also the porous structure of the electrode, thus leading to a trade-off relation on the proton and oxygen transport resistance. Yakovlev et al. investigated the ionomer coverage and thickness at different I/C ratios, ranging from 0.1 to 1, and they found that the increase in ionomer content leads to the increase in coverage until the system has a nearly full ionomer coverage when I/C ratio is low, while the increase in ionomer content leads to the increase in thickness when the I/C ratio is high [123]. As shown in Fig. 8c [88], the mean pore diameter of CCLs was measured to be 50.3, 40.3, and 29.8 nm for I/C 0.65, 0.8, and 0.95, and the corresponding porosity is 64.0%, 53.3%, and 51.4% respectively. To reduce the coverage and thickness of the ionomer, proton-conducting media such as polybenzimidazole have been incorporated at the catalyst sites, demonstrating positive effects [124]. The performance of fuel cells can be enhanced by selecting a suitable I/C ratio to optimize oxygen transport path based on ensuring the proton conduction. In addition, the equivalent weight (EW), the length and structure of sidechain, and/or backbone of the ionomer could be significantly related to the water content, main-chain molecular mobility, and degree of dispersion that directly impact the local oxygen transport in CCLs [125]. Low EW and short-side chain (SSC) ionomers due to their higher water uptake and higher proton conductivity enable substantial improvements in cell voltages particularly at higher operating temperatures (> 80 °C) and sub-saturated RH conditions (≤ 90% RH) (Fig. 8d) [126]. The SSC ionomer with increased amount of hydrophilic side chain favors greater phase separation due to higher water sorption, and this could promote the segregation interaction between the hydrophilic and hydrophobic domains and make a better oxygen transport path in ionomer [114, 127, 128].

The morphology and structure of the ionomer also will be destroyed seriously after chemical decomposition by the hydroxyl radical (·OH) during practical fuel cell operation. The ·OH is produced from hydrogen peroxide (H_2_O_2_), which results from the Pt-catalyzed reaction between the crossover of reactant gases (H_2_ and O_2_), or simply at the cathode, accompanying the oxygen reduction reaction (ORR) [129]. The decomposed fragments are the side chains of the ionomer polymer, and their repeat units gradually fall off, resulting in structural damage. The impact of alterations in the ionomer’s structure on proton conductivity is well acknowledged. However, the influence of these changes on oxygen transport has yet to be thoroughly investigated. It is suggested that the exercise of its main chain further reduces the hydrophilic and hydrophobic phase separation structure in the film and reduces the oxygen transport path. On the other side, several studies have elucidated the structural loss in ionomer films, indicating a reduction in thickness and led to a decrease in oxygen transport resistance. Further research is required for the relationship between ionomer degradation and oxygen transport process, which has significant implications for fuel cell durability especially in low or ultra-low Pt loading MEAs.

##### Carbon *Support*

Catalyst supports can be just as important as catalyst nanoparticles in determining overall MEA performance, which is related to catalyst utilization, mass transport, and durability. The carbon supports affecting the galvani potential of the catalyst and reduce its fermi level increases electron density and accelerate electron transport, thereby improving the ORR performance and stability of the catalyst [130]. Catalyst nanoparticles are uniformly distributed on the support conventionally to offer high ECSA [131, 132], and thus, the structure of CCLs and the TPBs are highly dependent on the support microstructure and morphologies.

The low surface area carbon (LSAC) such as XC-72 does not contain sufficient anchoring sites for catalysts, and thus, the Pt nanoparticles are easily shifted and further agglomerated together. The high surface area carbon (HSAC) has been developed to enhance the specific surface area of the carbon support. The numerous primary pores facilitate the prevention of –SO_3_^−^ groups-induced poisoning of the ORR kinetic activity for Pt nanoparticles. Oxygen transport mechanism for catalysts within the pores differs from that on the surface. Both oxygen transport resistance in ionomer (*R*_ionomer_) with the limiting effect of thickness, and in interior pores (*R*_interior_) with the  narrow openings (1–4 nm) and tortuous structures, exhibit the *R*_local_ for HSAC, as depicted in Fig. 8e [133]. On the side, the proton conduction is also limited by the increased transport path distance and insufficient penetration of the ionomer into the small pores, which hinders the effective contact between proton and interior Pt nanoparticles [49]. The development of higher active and stable catalyst supports must show optimized pore structure on the surface to be combined with the kinetic activity and the proton and mass transport (Fig. 8f).

The systematic effects of pore structure of HSAC on ionomer distribution and mass transport have been thoroughly investigated by researchers at General Motors. Ramaswamy et al. have performed a systematic analysis of the underlying microstructure of catalyst dispersed on various HSAC supports in terms of their pore size distribution. They found that the micropore (< 2 nm), mesopore (2–8 nm), and macropore (> 8 nm) surface areas of the carbon support are directly correlated with the local oxygen transport as well as the proton conductivity. The increase in the number of micropores (< 2 nm) is contribute to an elevated oxygen transport resistance due to the challenging transport process of oxygen through tortuous and narrow micropores, and the proton conduction is directly proportional to the number of macropores (> 8 nm) as it disrupts the network coherence of ionomer [115]. The adjustment of the Pt/ionomer interface through the pore structure of the carbon surface is widely believed to be an effective means, with mesopores ranging from 4 to 7 nm appearing as a promising target for future support development [116].

The surface chemistry of the carbon support is also important, and especially the functional groups will act as anchoring sites for metal catalysts and could alter the distribution of ionomers and impacts the structure of TPBs. Cheng et al. found that the carbon support EA with high degree of graphitization showed higher *ξ* relative to those for XC-72 and EC300J, which is ascribed to a lower concentration of oxygen-containing functional groups (Fig. 8g). It is hypothesized that the positive charge could cause a more homogeneous ionomer distribution as well as a more uniform ionomer film thickness on carbon surface, which results in a lower* R*_local_ [117]. It has been considered that the heteroatoms substitutional-doping method and functionalization could intend to engineering TPB when modifying physical and chemical properties of carbon supports.

Carbon supports degradation could also influence both bulk and local oxygen transport resistance. Generally, carbon supports can initially be oxidized to introduce surface oxygen-containing functional groups. The optimized content of oxygen-containing functional groups facilitates water and proton conduction, whereas an excessive amount of such groups is undesirable [61]. It could lead to decreased ionomer adsorption on carbon support, thus leading to the ionomer agglomeration and increasing the oxygen transport resistance at the TPBs. With the further degradation of carbon support, the carbon loss could lead to structure collapse, thereby increasing *R*_bulk_ in the CCLs.

#### Effect of Operating Conditions on Oxygen Transport

In PEMFC applications, such as automotive, a stack is expected to perform under a wide range of operating conditions including highly variable humidity, temperature, and pressure range. Researches have proved that the operating conditions make significant influence on thermodynamic performance [134], heat transfer [135], water transport [135–137], and catalyst durability [111, 138, 139]. In terms of the oxygen transport problem in low or ultra-low Pt loading electrode, the effect of operating conditions on oxygen transport is also apparent.

The unique nanophase-separation structure of ionomer films gives it a high sensitivity to water adsorption, making RH significantly influence both ionomer–water content and morphological. It has been experimentally proved by Shen et al. that the *R*_local_ decreases accompanied with the increase in the RH [87], where the volume fraction of water will increase in the ionomer and correspondingly, the fraction of hydrophobic PTFE will decrease. This is consistent with the gas permeability research that the oxygen permeability in water is four times higher than that in hydrophobic PTFE [140]. The gas permeability of oxygen here is the product of the dissolved oxygen concentration (*C*_O2_) relating to the adsorption step and oxygen diffusion coefficient (*D*_O2_) relating to the diffusion step in “adsorption-diffusion” model. Oxygen is considered to dissolve predominantly in the hydrophobic domain, while the predominant pathway for oxygen diffusion is through hydrophilic domains [91]. The increasing trend of *D*_O2_ is much higher than the slight decrease in *C*_O2_ under the condition of the same increase in RH [141], thereby causing the oxygen permeability increases along with the increased water content.

The operating temperature plays an essential role on ionomer structure, oxygen diffusion efficiency in ionomer and pore structure, etc. Nonoyama et al. suggested that especially at lower temperature the local oxygen transport is dominant [40], as the oxygen permeation resistance of the ionomer is a much stronger function of temperature than molecular or Knudsen diffusion. This results in about a 2.4 times increase in oxygen transport resistance in ionomer from 80 to 40 °C. Shen et al. demonstrated that *R*_local_ decreases from 0.89 to 0.38 s cm^−1^ with the increase in the operating temperature from 40 to 80 °C, and they believed that the increase in the operating temperature will cause the decrease in *R*_local_ via both the PTFE and water paths [87]. The increase in the operating temperature results in the increase in the water vapor partial pressure (P_H2O_) under the same RH condition, which enlarges water channels in ionomer film. In addition, the operating pressure could influence *R*_local_ due to the as-induced concentration of oxygen *c*_O2_. Shen observed that the* R*_local_ increases ~ 81% as the *c*_O2_ increases from 0.614 to 0.818 mol cm^−3^, which could be related to the limitation of oxygen transport due to saturated adsorption at gas/ionomer interface. Therefore, the control of operating conditions is crucial. Selecting appropriate operating conditions can significantly enhance performance and durability.

## High Oxygen Transport Electrode Design

The bulk and local oxygen transport in CCLs is highly dependent on the microstructure of CCLs, including the pore structure, and TPBs structure related to the key materials properties. The mechanism of oxygen transport described above could provide guidance for the design of electrode structure. The insufficient stability of conventional CCLs, consisting of carbon-supported catalysts and ionomer films, also poses a significant barrier for reducing Pt loading. Recent developments in migration strategies for reducing oxygen transport resistance in CCLs have been reviewed to achieve high power density, durability, and efficiency. Novel electrode structures with excellent pore structures that result in satisfactory proton and mass transport characteristics have been reported. Furthermore, emphasis has been placed on designing key materials related to electrode structure, such as carbon support and ionomer, to enhance durability particularly under high current densities.

### Novel Structure Design

**Fig. 9:**
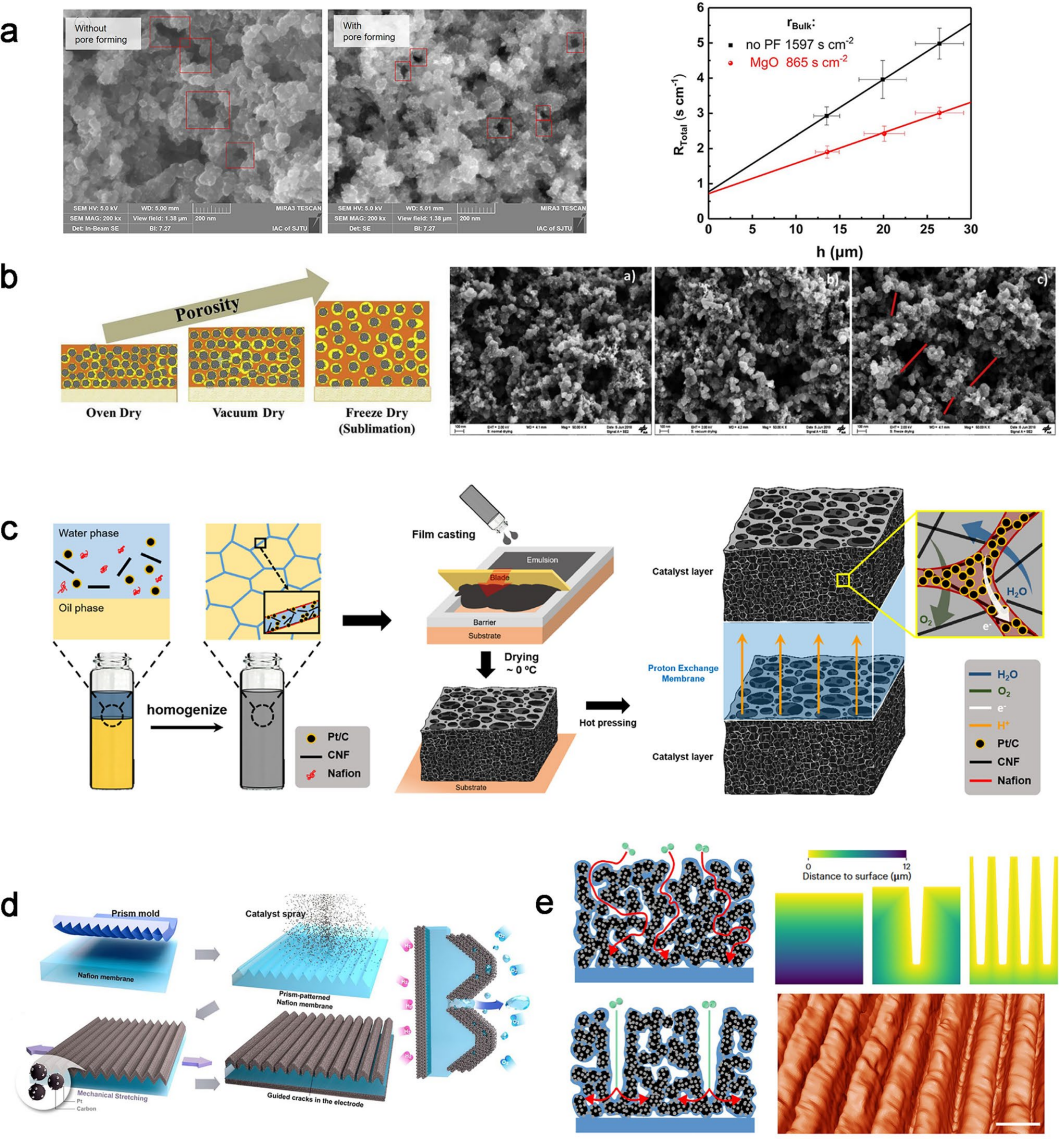
Novel structure designs for CCLs in PEMFC. **a** SEM images for MgO pore-formed and original CCLs (left), and Plot *R*_Total_ vs. *h* for both MgO pore-formed and original CCLs (right) [71]. Copyright 2019 The Electrochemical Society. **b** CCL structure comparison for oven dry, vacuum dry and freeze dry [143]. Copyright 2019 Elsevier B.V. **c** Scheme of the emulsion-templated fabrication process and structural features of the resulting CL [144]. Copyright 2021 American Chemical Society. **d** Schematic illustration of guided crack generation in an electrode by stretching the prism-patterned MEA [145]. Open access. **e** Schematics showing oxygen transport through flat and grooved electrodes, and Euclidean distance from the interior of the electrode to the surface, and reconstructed nanoscale computed X-ray tomogram of a grooved electrode [42]. Open access

The pore-forming templates, for example, polymer-based sub-micrometer beads or mental oxidation, are mixed into the catalyst ink to increase the porosity of CCLs and facilitate oxygen transport. Ammonium carbonates, lithium carbonate, ammonium sulfonate, or ammonium oxalate can be used as pore former after a simple removed method by a heating process and lead to low resistance for gas diffusion [69]. However, this heating process could affect the membrane and catalyst activity. Cheng et al. modified the CCLs structure using magnesium oxide (MgO) as the pore-forming agent, and it was removed via the acid washing after the porous CCLs formed. The porosity increases from 53% to 65%, and the most probable pore diameter enlarges from 40 to 85 nm after pore-forming (Fig. 9a). The enlarged pore structure significantly decreased *R*_bulk_ from 1597 to 865 s cm^−2^ [71]. The mechanism of pore size distribution on *R*_bulk_ is further explored by introducing nano-calcium carbonate (CaCO_3_) with varying particle sizes. A “sphere-pipe” model for bulk oxygen transport in CCLs was provided, where oxygen molecules need to first diffusion across the narrow pipes inside the catalyst agglomerates and then diffuse in large secondary pores [142].

However, the advanced pore structures from these pore-forming structures accompany a complicated and expensive manufacturing process. Therefore, a simpler and more efficient method is needed to prepare an orderly and stable mesoporous structure in the CCLs. Free casting or ice templating method is a particularly versatile technique that has been applied extensively for the fabrication of well-controlled porous materials based on carbon materials, endowing them with novel properties and broadening their applicability [146]. As shown in Fig. 9b, Talukdar et al. make the electrodes sublimation under 0.1 mbar with the use of liquid nitrogen as coolant for the CCLs. The porosity displays a distinct peak at 90 nm and a high total pore volume in the macropores region (> 100 nm) and shows an increased ECSA by 1.5 times. They also found that the ice templating method constructs a homogeneous geometry of the pores, which strongly influenced the interaction between ionomer and carbon support and improved ionomer distribution, endowing a less proton and oxygen transport resistance across the active sites [143].

In addition, emulsion template using high internal phase emulsions (HIPEs) comes into effect in the pore forming in CCLs with simple operation and high mass transport efficiency. As shown in Fig. 9c, oil droplets in the slurry are stabilized by the hydrophilic surrounding phase of the catalyst, ionomer, and solvent. After evaporation of the liquid components, macropores were generated due to the removal of the liquid, and the integrity of the ionomer and catalyst remained as a frame with mesopores, resulting in a multiscale porosity [144]. The one-step formation of controlled channels for CCLs is considered to exhibit high performance via improved oxygen transport. The guided cracks for CCLs generated by stretching a catalyst-coated PEM with high plastic deformation can play a similar role as oxygen transport channels [147]. As demonstrated in Fig. 9e, the formation of microprism-patterned arrays through mechanical stretching using the concentrated surface stress can result in a reduction of membrane resistance, as well as an enhancement in oxygen transport [145]. Further, the grooved electrode proposed by Lee et al. is separated by grooves (void channels) that could form the oxygen transport channels even the ionomer content is high (Fig. 9d). This structure provides up to 50% higher performance than conventional electrodes under standard operating conditions [42]. It is noted that the partial catalyst disconnection with GDL and the bare PEM without contact with the CCL would increase as the width of generated cracks expands with the cracks or grooves; thus, an optimized morphology on the electrode is necessary to balance oxygen and water transport while addressing the limitations of electrochemically active sites.

Although highly efficient pore structures have effectively enhanced oxygen transport pathways in the CCLs, research on novel-constructed pore structures in the literature often lacks stability during long time operation. For example, carbon corrosion especially in high potential operation conditions often makes a reduction in the porosity and thickness in conventional CCLs. Therefore, implementing effective strategies is crucial for ensuring the stability of these structures and warrants increased attention moving forward.

### Carbon Support Design

**Fig. 10:**
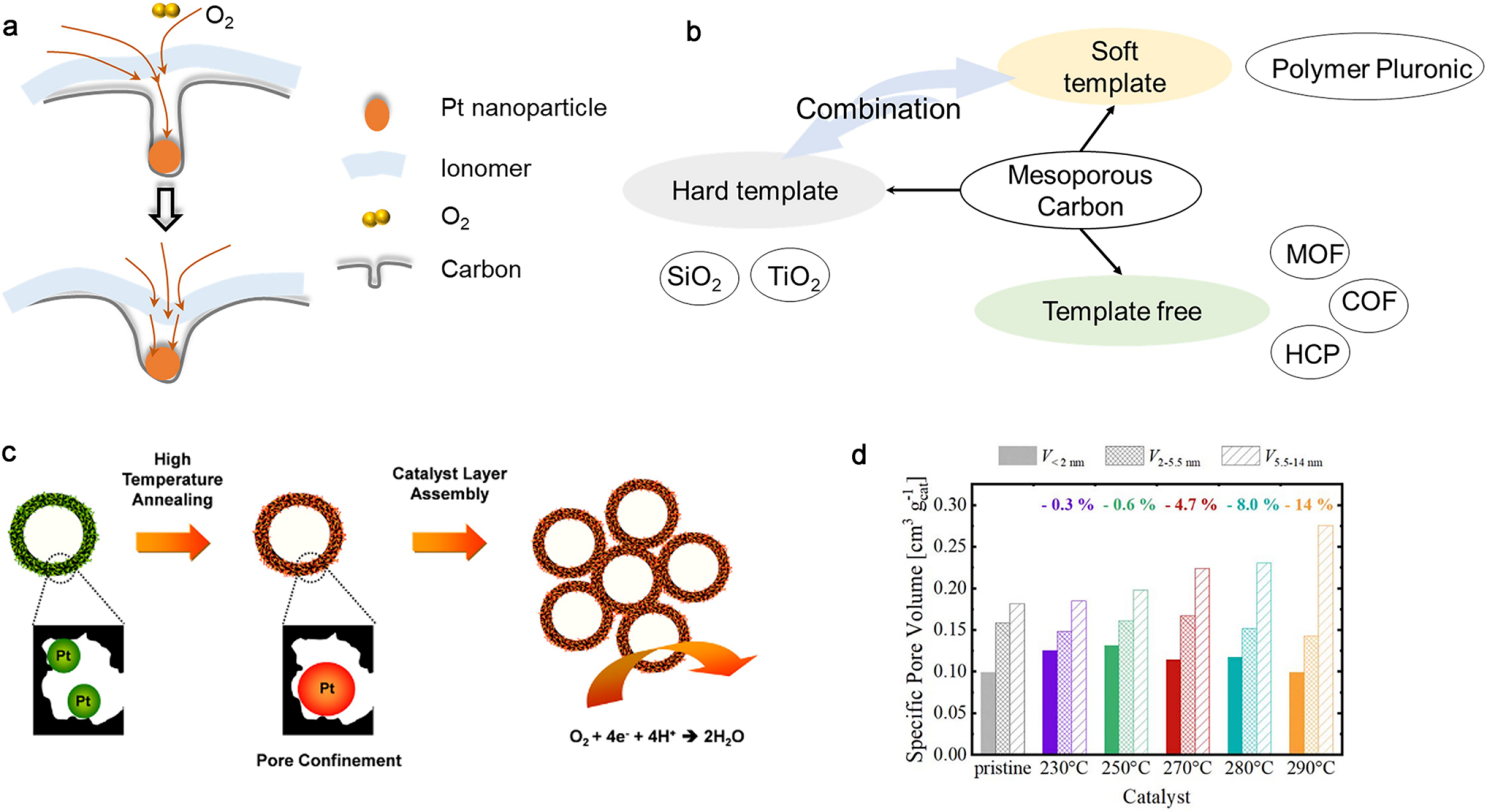
Strategies for carbon support design. **a** Oxygen transport in original and accessible pores on carbon surface. **b** Summary of fabrication method for mesoporous carbon. **c** Schematic model of Pt encapsulation by pore confinement [151]. Copyright 2012 American Chemical Society. **d** Differential pore size distribution determined by N_2_ physisorption for the catalysts oxidized in the tube furnace at various temperatures for 12 h [44]. Open access

The physical–chemical properties of the carbon supports, including pore structure, degree of graphitization, and surface functional groups, can have a large effect on the performance of the fuel cell, especially relating to the structure of TPBs. Although many new support materials have emerged, they have poor electrical conductivity and are difficult to support catalysts [148–150]. Carbon materials are the most widely used catalyst support nowadays for high ORR activities. The adjustment of the porous microstructure and the morphologies for better ionomer/carbon interface obtain the improvement of oxygen transport. Optimizations should be put forth for accessible porous carbon featuring an internal pore structure in which Pt nanoparticles would be protected from ionomer contact to allow for high ORR activities and durability through time operation. Moreover, as mentioned in Sect. 2.4.2 the pore structure on the HSAC plays a vital role in the activity and the local oxygen transport process, and thus, effective carbon regulation should be proposed to have properties such as ultrahigh surface areas, large pore volumes, and enlarged the pore size (Fig. 10a).

The synthesis of mesoporous carbon materials in electrode is divers, including hard template, soft template methods, and template-free-based methods, as shown in Fig. 10b [152, 153]. Mesoporous carbon materials prepared by hard template, such as the mesoporous silica and mental oxidation, exhibit superior performance compared to primarily microporous carbon supports in terms of enhancing Pt accessibility and facilitating proton and mass transport during electrochemical reactions (Fig. 10c) [151, 154, 155]. However, the hard template method is far fewer hard templates available for use and the procedure is complex and time-consuming. The soft template method makes surfactant molecules, and guest species co-assemble into ordered mesostructured composites [156]. The combination of soft and hard templating routes can prepare pore structures of different scales to meet the requirements of oxygen and liquid transport in CCLs. The template free method is also widely used in the preparation of mesoporous carbon supports, which mainly include metal complex such as metal organic frameworks (MOFs), hypercross-linked polymer (HCP), and covalent organic framework (COF). They can directly yield carbons within template because they consist of carbon precursor and template at the same time, where the mesopore voids stem from the aggregation of nanoscale building blocks and play a role of catalyst in itself [157–159]. The application of a variety of preparation methods to synthesize mesoporous carbons materials has been described in detail in many excellent reviews and reports, which highlights the ORR activity improvement and the durability of catalyst and carbon support.

With the commercialization of porous carbon used in PEMFCs, Ketjenblack EC 300J has been widely applied and is one of the most prominent carbon supports nowadays. The surface area of Ketjenblack EC 300J is 807.9 m^2^ g^−1^, and the most probable pore diameter is 3.6 nm, which could trap catalysts amounts to 60%–70% in internal pores [160] and show excellent oxygen reduction reaction (ORR) kinetics activities, but hinders the local oxygen transport at the TPBs due to small pore size. The modifications of pore structure of the commercial porous carbon have achieved. Gasteiger et al. develop the carbon pore textural structure of Ketjenblack EC 300J by subjecting it to heat treatments rang in an air/Ar mixture; therefore, the pores in the carbon support could be opened up by the immediate vicinity of Pt nanoparticles through air. The modified catalyst exhibits a strongly reduced *R*_NP_ of 0.46 ± 0.01 s cm^−1^ compared to 0.56 ± 0.01 s cm^−1^ for the original catalyst (Fig. 10d) [44]. Carbon etching in NH_3_ also makes sense of the structure modification on the carbon surface, where the etching rate is different between the graphitic crystallites and the disordered matrix phase [161]. One approach to modify the commercial carbon support was recently presented by Ott et al. that the Ketjenblack EC 300J was heat treated at elevated temperature (200, 400, and 600 °C) using NH_3_ promoting the morphology and structure of carbon surface. It is explained that carbon etching at high temperatures forms humic substances in the presence of NH_3_, resulting in opening of micropores and an increase in its external surface area, thereby reducing the oxygen transport resistance [41]. Moreover, Zhang et al. introduced perchloric acid (HClO_4_) as a pore-forming agent to modify the pore structure of carbon nanospheres using a local ablation method. The modified catalyst shows a significantly reduced *R*_NP_ from 0.56 to 0.46 s cm^−1^ [162].

Overall, it is widely acknowledged that the microstructure of carbon supports plays a crucial role in oxygen accessibility. And it also significantly influences the distribution of ionomers, while the interactions between porous carbon supports and ionomer thin films, as well as the degradation mechanisms affecting the porous structure, require further investigation to ensure efficient and stable TPBs.

### Ionomer Design

**Fig. 11:**
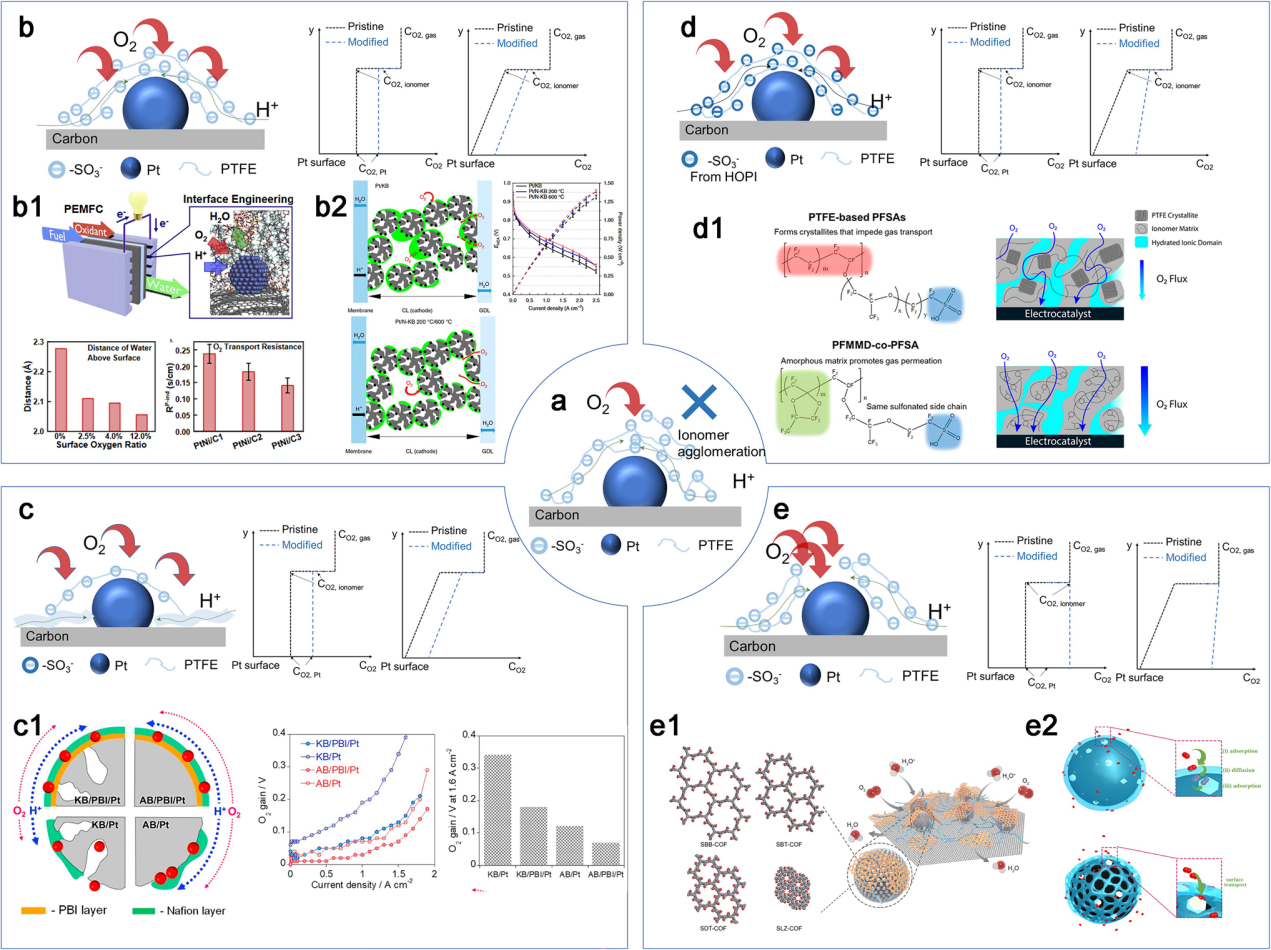
Design of ionomer morphology and structure. **a** Illustration of traditional inhomogeneous ionomer distribution at the TPB. **b** Illustration of uniform distribution of ionomer at the TPB and oxygen transport mechanism; **b1**:TPB engineering based on oxygen-containing functional groups [163]; Copyright 2025 Elsevier Inc. **b2**:ionomer distribution on N-modified carbon and their polarization curves [41]. Copyright 2025 Springer Nature Limited. **c** Illustration of reduced ionomer at the TPB and oxygen transport mechanism; **c1**: illustration of PBI on carbon surface and oxygen gain [164]. Copyright 2020 Elsevier B.V. **d** Illustration of HOPI at the TPB and oxygen transport mechanism; **d1**: traditional ionomer such as Nafion (top) consist of a semicrystalline PTFE matrix and a sulfonated pendant side chain [165]. Copyright 2020 American Chemical Society. **e** Illustration of pore form of ionomer films at the TPB and oxygen transport mechanism; **e1**: schematic illustration of the gas and proton transfer in Pt/C@COF-Nafion [166]; Copyright 2022 the authors. **e2**: transport mechanism of local oxygen transport in original and pore-form nanoporous ionomer film [59]. Copyright 2022 Published by Elsevier B.V

Oxygen transport in ionomer films with the unique phase separation structure is considered to obey “solution-diffusion” model, which is the primary factor influencing *R*_local_. The most commonly used polymer in CCLs nowadays is PFSA ionomers, such as Nafion in Dupont Company. It gives high proton conductivity, but low oxygen permeability, thereby reducing cell performance in high current density. The unexpected inhomogeneous ionomer distribution on the carbon support/catalysts surface in traditional CCLs as shown in the middle of Fig. 11a has garnered notable attention. Considerable efforts have been devoted to improve the oxygen transport ability in CCLs without satisfying proton conduction, including (1) make the ionomer films uniformly distributed on carbon and catalyst surface (Fig. 11b); (2) reduce ionomer coverage in CCLs (Fig. 11c); (3) fabricate high oxygen permeability ionomers (HOPI) through structure design (Fig. 11d); and (4) form pore structure in ionomer films (Fig. 11e).

The uniformly distributed ionomer films show a thinner thickness and lower oxygen concentration gradient than ionomer aggregations on the carbon and catalyst surface. As illustrated in Fig. 11b, for low current density, the oxygen demand is minimal and is not substantially hindered by the ionomer films, leading to a consistent oxygen concentration throughout the ionomer. Consequently, a reduction in the thickness of the ionomer films results in a lesser decrease in oxygen concentration from the gaseous phase, thereby enhancing the availability of oxygen at the Pt surface. For high current density, a decrease in the thickness of the ionomer films leads to an increased oxygen flux toward the Pt surface. The distribution of ionomer at TPBs is mostly related to properties of the carbon surface, such as the functional groups.

Oxygen-containing functional groups has been proven to make the mental precursor more accessible due to the more hydrophilic surface chemical properties and make an adjustment of pH as the catalyst preparation and is considered nucleation sites of metal crystals [167, 168]. However, the excess oxygen-containing functional groups could result in inhomogeneous ionomer distribution because the more hydrophilic property and the negative charge on carbon surface could decrease the interaction between carbon surface and ionomer [169]. A desired content of oxygen-containing surface functional groups plays a controlling role in the mass transport, where both the hydrophilic side chains and hydrophobic backbones can interact with the catalyst for proton, oxygen, and water transport. Zhao et al. have found that the *R*_NP_ is decreased since there is a more uniform distribution of the ionomers when the oxygen contents are 2.4% and proposed that moderate contents of oxygen-containing functional groups can improve the overall performance of the fuel cell, as shown in Fig. 11b1 [163].

The amide/imide/lactam functional groups (–NH_*x*_) have been widely functionalized on the carbon support for optimization of TPBs by making a homogeneous ionomer distribution, where –NH_*x*_ could ionically interact with the ionomer’s sulfonic groups (–SO_3_^−^) through coulomb effect [170, 171]. Orfanidi et al. functionalized the surface of a commercially available carbon with –NH_*x*_ groups and found that the performance improvement between Pt/V-NH_x_ and Pt/V cannot be solely attributed to the difference of microporosity of the carbon supports, but also related to a more homogeneous ionomer distribution on the NH_*x*_-functionalized carbon support [172]. However, the –NH_x_ groups proved insufficiently stable to sustain the suppression of local oxygen transport losses. Ott et al. introduce balanced quantities of sp^2^ pyridinic/pyrrolic/graphitic N functional groups that interact with the ionomer chains and promote their homogeneous spatial distribution. As shown in Fig. 11b2, the homogeneous ionomer distribution makes the Knudsen oxygen transport resistance in micropores decreased, thus leading a high performance under ultra-low Pt loading MEAs. A substantial enhancement of stability was also hypothesized as a consequence that the N groups act also as anchoring sites toward Pt during the Pt deposition process and do not interact only with the ionomer [41]. The sulfonic acid functional groups have often been emerged as the covalent functionalization in the surface of graphite carbon surface as a powerful strategy to produce high stability for catalyst/carbon interface and lead to a significant increase in the hydrophilicity, which is helpful for the catalyst dispersion [173–175]. However, this functional group does not facilitate the distribution of the ionomer; rather, it may lead to ionomer agglomeration due to its negative charge. Li et al. found that the positively charged carbon support with –NH_3_^+^ would attract with the negatively charged –SO_3_^−^ groups in the side chain of the ionomer particles, and the uniform ionomer/catalyst interface is spontaneously formed [43].

Introducing additional proton transport pathways on the carbon surface, while maintaining oxygen transport efficiency, represents an effective strategy to mitigate oxygen transport resistance within the ionomer. This method also reduces the thickness of the ionomer, and the mechanism for the reduced *R*_local_ is shown in Fig. 11c. Polybenzimidazole (PBI) was initially reported by Nakashima et al. as a dispersant for carbon nanotubes via a non-covalent polymer wrapping mechanism. The individual dissolution of carbon nanotubes and the effective reinforcement of PBI are attributed to *π*–*π* interactions [176]. Among the variety of PBI derivatives reported to date, pyridine-containing PBI not only possesses a significantly higher proton conductivity even without humidification due to its higher acid doping ability and better mechanical properties, but also serves as a glue for immobilizing Pt nanoparticles onto the graphite carbon support surface without any strong oxidation process [177, 178]. Berber et al. designed a novel polymer electrolyte membrane fuel cell (PEMFC) catalyst based on double-layer-polymer-coated carbon nanotubes as the catalyst support, where the polymers used for the double-layer coating are PBI and Nafion (Fig. 11c1). The interactions between ionomers and PBI-coated carbon surfaces can realize the formation of well-balanced protons and an oxygen transport network on carbon surface [124, 164, 179]. Further, in the past decade, thanks for the excellent proton conduction as well as the high oxygen permeation, ionic liquids (ILs) have shown to be promising additives to the catalyst layer to enhance oxygen reduction reaction and permeation in CCLs [180–182].

The mechanism of HOPI specifically engineered from side chains and backbones of ionomers, is shown in Fig. 11d [183]. For low current density, the increased oxygen solubility facilitated by HOPI utilization results in a smaller decrease in oxygen concentration from the gaseous phase, thereby enhancing the availability of oxygen at the Pt surface. For high current density, the enhanced solubility and diffusivity of oxygen through the ionomer contribute to an overall increase in the oxygen flux toward the Pt surface. The ring structure with high oxygen permeability has been polymerized in the backbone of the ionomer, increasing the local oxygen transport in ionomers without sacrificing the proton conductivity. Rolfi et al. synthesized a new amorphous ionomer from the modification of Aquivion PFSA with a steric hindered monomer (2,2,4-trifluoro-5-trifluoromethoxy-1,3-dioxole, MDO) in the backbone and have found that the cast membrane demonstrate a 20% higher oxygen permeability of the new ionomer compared to conventional PFSA [184]. Katzenberg et al. present a novel ionomer incorporating a glassy amorphous matrix based on a perfluoro-(2-methylene-4-methyl-1,3-dioxolane) (PFMMD) backbone. It slightly reduces proton conductivity because of the reduced swelling under hydration, while significantly improving gas permeability as the PFMMD disrupts matrix crystallinity in the ionomer as shown in Fig. 11d1, and achieved 22% higher current density at 0.25 V than the device employing Nafion [165]. Jinnouchi and Braaten et al. also demonstrate that a HOPI incorporating a ring-structured monomer, perfluoro-(2,2-dimethyl-1,3-dioxole) (PDD), significantly enhances the local oxygen transport at the TPBs due to the reconstruction of ionomer/Pt interface [185, 186]. In addition, the structure exhibits excellent performance under different RH. In the low humidity, the hydrophilic domains shrink, causing the ionomer’s structure to be dominated by the hydrophobic backbone structure [184]. In the high humidity, the ring structure can reduce swelling, which could hinder the swollen ionomer filling in the pores of CCLs [186–188]. The encouraging result motivates the structure design of ionomers to improve the oxygen permeability and promote the local transport required in the PEMFCs. Another effective and feasible scheme is to add additives with high oxygen permeability to increase oxygen transport properties without sacrificing proton conduction. Based on a parallel two-nozzle system with separated solution reservoirs, polydimethylsiloxane (PDMS) with high gas permeability, chemical stability, and hydrophobicity was employed to protect the CCLs by Yeon et al. The novel ionomer structure with optimal amount of PDMS improves the performance through the high oxygen transport and decreases the degradation of carbon corrosion due to more sensible water management [189].

The design of ionomer structure coated on the Pt/C surface in CCLs is another novel and promising focus of HOPI’s research, which could result in a more efficient and durable TPB for oxygen transport and ORR. Recently, facile approaches have been introduced to prepare ionomer films coated on the Pt/C surface, which make a positive influence on the oxygen and proton transport in CCLs. As shown in Fig. 11e, the pore in ionomer films breaks the transport pathway based on “solution-diffusion” model, where the oxygen can reach the active sites without the ionomer. Zhang et al. optimized the TPBs by incorporating ionic covalent organic framework (COF) nanosheets into Nafion for enabled the proton transport and promoted oxygen permeation (Fig. 11e1), where the intrinsic mesopores of the frameworks make *R*_NP_ decrease 60% [166]. Cheng et al. successfully regulate ionomer thin films with nanoscale pores using polyvinyl alcohol (PVA) as a sacrificial pore-forming agent, which can well multiply TPBs and provides more oxygen reduction reaction sites without the “adsorption-diffusion” process (Fig. 11e2). It makes the *R*_local_ reduce from 0.37 to 0.08 s cm^−1^, corresponding to 78% [59]. Recently, Chen et al. designed a noncovered catalyst/ionomer interfacial structure, which employs the ionomer covered Pt/C catalyst surface as proton transport channels and ionomer noncovered Pt/C catalyst for oxygen and water transport channels in the CCL. This structure provides higher peak power density of the PEMFC with the noncovered catalyst/ionomer 77% and 67% higher than the covered-type of oxygen and air conditions, respectively [190].

The strategies for decreasing *R*_lcoal_ in CCLs based on ionomer morphology and structure have been studied in depth, while the coupled transport of oxygen and protons was further need to optimize to adapt to the gradually increasing temperature and decreasing RH in applications. At the same time, the durability of the interface is also worth considering.

## Insight into Oxygen Transport in PEMWEs

The above has been detailly reviewed the oxygen transport process in CCLs of PEMFCs, including the bulk oxygen transport in pore structure and the local oxygen transport at “gas/ionomer/catalyst” TPBs, and the directions of performance optimization from the aspects of novel structure, carbon support, and ionomer design are also proposed. Electrochemical gas evolution reactions are also of vital importance processes for PEM water electrolyzes (PEMWE), and the mass transport including the reactants (liquid H_2_O) and products (O_2_ in the anode and H_2_ in the cathode) resistance also occurred in CLs consists of catalyst and ionomer. The performance is limited due to oxygen transport for the anode reaction, leading to insufficient reactant supply at higher current densities [191]. The similarity in MEA structure between PEMWEs and PEMFCs raises concerns regarding oxygen transport in PEMWE, prompting us to draw inspiration from the more advanced research conducted on PEMFC, because the forms of reactants (O_2_ and H_2_ in PEMFC and liquid H_2_O in PEMWE) and products (gaseous or liquid H_2_O in PEMFC and O_2_ and H_2_ in PEMWE) are different. Depending on the amount of oxygen produced, the flow behavior can vary from single-phase (with unsaturated oxygen generation) to various forms of two-phase flow (with oxygen bubbles) and encountered new challenges. The research of mass transport in WE should be paid more attention to the two-phase flow and bubble dynamics.

### Significance for Oxygen Transport in PEMWEs

The occurrence of OER in the anode is difficult due to a four-electron transfer process where two water molecules are oxidized, generating four protons (H^+^) and one oxygen gas molecule. The Ir-based catalysts typically demonstrated high activity and stability in acidic environments are the most widely employed anode catalyst [192–194]. The high cost and limited reserve of novel metal drive the development of their oxides [195], alloys [196], and Ir-based catalysts in various structural forms [197, 198], such as perovskites, pyrochlores, and single-atom dispersions [199]. In addition, to decrease the loading of scarce and expensive Ir-based catalysts and maintain good catalytic performance, the successful method is to anchor catalyst nanoparticles on a high-surface area conductive support. Cerium oxide [200], titanium oxides [201], manganese dioxide [202], nano-metal diborides [203], or other materials possessing good electrical conductivity, large specific surface area, and high resistance from acid corrosion and oxidative decomposition are used to support catalyst nanoparticles for realizing low-Ir loading PEMWE. Owing to the strong catalyst-support interactions that tune the electronic structure of Ir and its adsorption energy to OER intermediates, the supported catalysts activities are often comparable to or even superior to that of pure Ir/IrO_x_ nanoparticles. Recently, Shi et al. introduced a ripening-induced embedding strategy that securely integrates the Ir catalyst into a cerium oxide support. A PEMWE using this catalyst achieves a cell voltage of 1.72 V at a current density of 3 A cm^−2^ with an Ir loading of just 0.3 mg_Ir_ cm^−2^ and a voltage degradation rate of 1.33 mV h^−1^ [194].

**Fig. 12:**
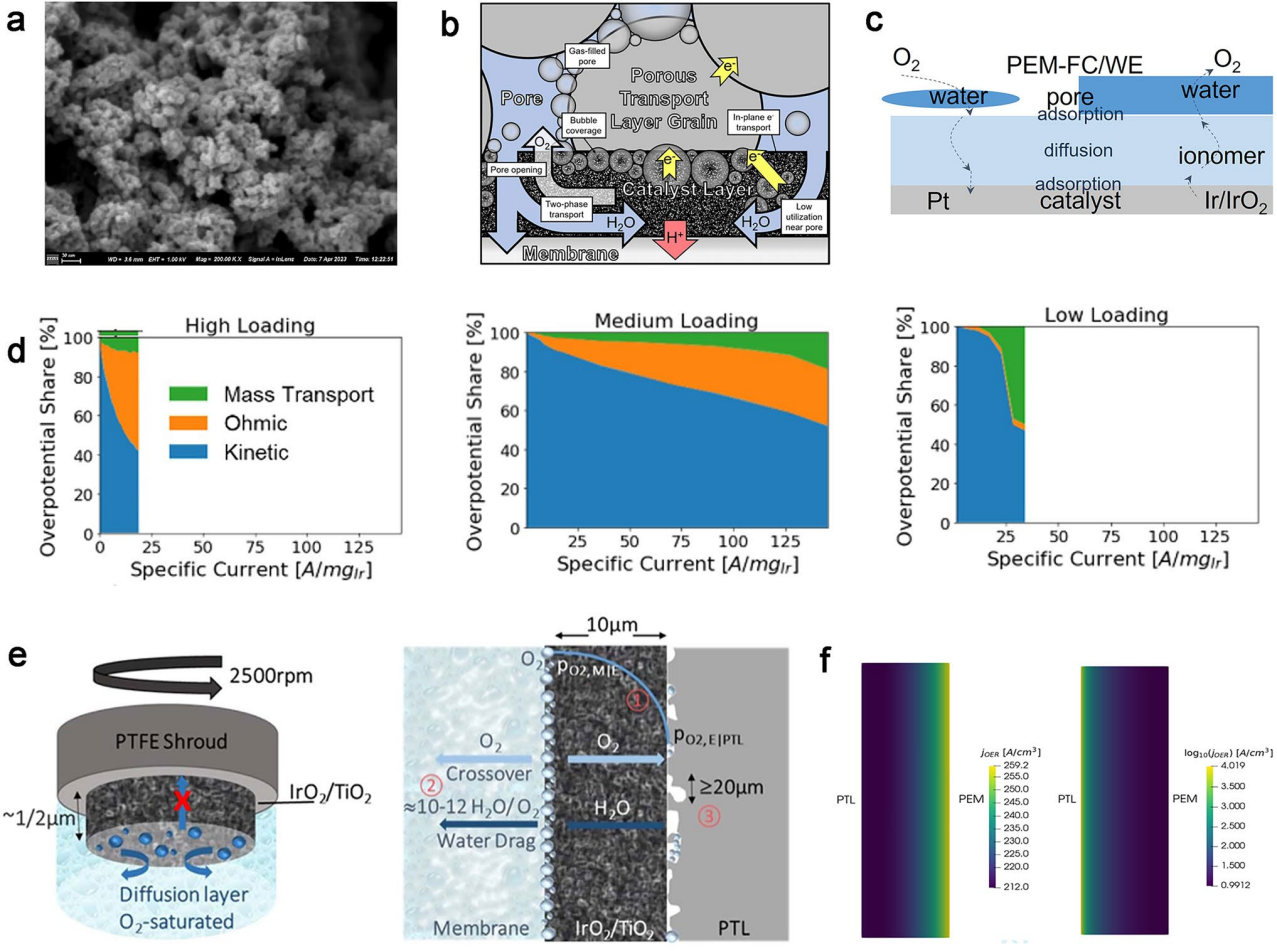
Oxygen transport behavior in the anode of PEMWE. **a** Typical SEM image for the ACL. **b** Depiction of proton and mass transport phenomena in ACL and PTL [205]. Copyright 2020 The Author(s). **c** Comparison for the oxygen transport at the TPBs between PEMFC and PEMWE. **d** Overpotential share in high, medium and low catalyst loading [206]. Copyright 2020 American Chemical Society. **e** Scheme of the oxygen bubbles in RDE (left) and MEA (right) measurements [207]. Copyright 2021 The Author(s). **f** OER distribution for an Ir ACL (left) and an IrO_*x*_ ACL (right) [208]. Open access

The structure of the CLs in PEMFC and PEMWE exhibits both distinct similarities and differences overall. For the CCLs in PEMFCs, the Pt catalyst with a size range of approximately 2–5 nm is uniformly loaded on the carbon supports with the particle size of ~ 50 nm. These nanoparticles are coated with thin ionomer films with the thickness ranging from 5 to 10 nm. The electrode exhibits a wide distribution of pore sizes, ranging from tens to hundreds of nanometers. These pores can be categorized into primary pores (< 30 nm) and secondary pores (30–150 nm), which play distinct roles in local and bulk oxygen transport processes, respectively. For PEMWE, the commonly used Ir/IrO_*x*_ catalysts with a size of approximately 2–5 nm and ionomers are mixed to form agglomeration as shown in Fig. 12a, where the pore structure constitutes the main channel for oxygen and water transport. It is worth to pursue the development of cost-effective catalysts as a pivotal step toward achieving the commercialization of PEMWEs, while at high current densities, the existence of oxygen bubbles could lead to serious mass transport problem. Due to the covering of bubbles in the electrode structure, the reactant water cannot reach the reaction site.

The reactant water initially transported from the inlet of the flow fields, subsequently passing through the PTL before reaching the catalyst sites in ACL. Meanwhile, the produced oxygen is expelled first within the ACL and then through the PTL and flow fields [204]. The proton and mass transport process in the ACL and porous transport layers (PTL) of PEMWE is illustrated in Fig. 12b.

Oxygen produced at the catalytic sites undergoes a complex transport process in CL. Both bubble oxygen and dissolved oxygen are existed in the ACL. The pathways of oxygen transport in CCLs in PEMFC and ACLs in PEMWE are compared in Fig. 12c, where both two devices exclude the interface of catalyst, ionomer, gas, and water. The oxygen transport path forms a circular loop, and the pore structure of ACLs in PEMWE is filled with reactant water, making the TPB as the “catalyst/ionomer/water” interface, while there exists slight production water for CCLs in PEMFCs, making the TPBs as the “catalyst/ionomer/gas” interface.

The “CL/PTL” interface is another important structural impact factor influencing not only the ohmic, kinetic overpotential, but also the mass transport overpotential in PEMWEs [209]. Three regions for “CL/PTL” interface have been summarized as (i) the area in the open pore space, (ii) the area beneath the contacted PTL, and (iii) the interface between the pore and solid. Electron transport is limited by the in-plane electrical conductivity, while under the contacted solid, mass transport of oxygen and water is hindered [210]. Thus, the CL/PTL interface needs to consider the balance between contact area and oxygen transport possibility [211, 212]. The oxygen bubble transport in PTL is a complex two-phase flow process involving pressurization and penetration. Following these phases, the bubbles can traverse along low-resistance pathways while being impeded by high-resistance routes. The pore size [213] and wettability [214] could significantly influence the resistance encountered during bubble transport. After detaching from the PTL and flow field interface, bubbles disperse within the water flow in the flow field. At low current densities, the bubbly is shown as flow regime within the flow field owing to the lower frequency of bubble generation and smaller bubble sizes. Conversely, at high current densities, the increased frequency of bubble formation results in slug flow, where gas slugs fill the diameter of the channel. Ultimately, with increasing gas production, the slug flow evolves into an annular flow regime.

Many works have focused on the flow behaviors in flow fields [191, 215–217] and PTLs [204, 213, 218] to find out the relationship between oxygen bubbles behavior and performance, while the transport issues in ACL are still lacking. Oxygen accumulated in ACLs has an overall negative effect on the reaction, which is more significant due to the lower catalyst loadings (< 0.5 mg_Ir_ cm^−2^) and higher current density (e.g., 5 A cm^−2^) operation as shown in Fig. 12d, because the reaction at the unit catalytic site is more intense and more bubbles are produced [206]. This issue arises when the rate of oxygen production exceeds the capacity for efficient oxygen transport away from the local sites, resulting in higher oxygen transport resistance. As shown in Fig. 12e, the accumulation of microscopic oxygen bubbles in the pores of the catalyst layer during the OER takes place in both RDE and MEA measurements [207]. Due to the structural differences between RDE and MEA, it is now well established that oxygen transport process obtained from RDE measurements differs from that of in a MEA in a PEMWE under similar operating conditions [219]. The bubble removed motivation is considered to be the main source of this discrepancy. Within the RDE measurements with no positive pressure gradient from the disk–electrolyte to the catalyst–electrolyte interface, the oxygen can only be removed to by diffusion within the CL or by convection in the radial direction at the outer surface of the CL into an oxygen-saturated electrolyte. Within the ACLs in MEA measurements, the oxygen local partial pressure at the reaction sites must increase, resulting in a pressure gradient from the PEM/ACL interface to the ACL/PTL interface to enhance the removal of oxygen, thereby minimizing the accumulation of oxygen within the CLs [207]. This uneven gas distribution also greatly affects the performance of the MEA, especially at low catalyst loading conditions. Based on the high-speed microscale visualization system, Yu et al. found that oxygen bubbles could be observed accumulating at the reaction interface, limiting the active area and increasing the cell impedances [220]. In addition, the catalyst Ir or IrO_*x*_ showing different effective protonic and electronic conductivities could lead to varying OER reaction distributions of products in ACL, where the reaction could be concentrated at either the PTL or the membrane. The Ir-based ACL has an electronic conductivity that is almost ten times that of the protonic phase, whereas the electronic conductivity of the IrO_*x*_-based ACL is three orders of magnitude lower than the protonic. Moore et al. presented a 2D macro-homogeneous model describing the MEA of a PEMWE as shown in Fig. 12f. They found that the reaction in the IrO_*x*_ ACL occurs close to the PTL, and any evolved oxygen can quickly escape to the PTL and be removed in the channel. In contrast, the gas is evolved close to the membrane in Ir-based ACL [208].

To overcome the overpotential associated with the gas evolution reactions, it is essential to understand the structure of pore and “ionomer/catalyst/gas” interfaces in ACLs and how oxygen behaviors influence this.

### Oxygen Transport Resistance Characterization of PEMWEs

Due to the complexity of the electrode structure and the uncertainty of oxygen bubbles in the anode, it is difficult to quantify oxygen transport losses. The Tafel fitting method is usually used to analysis the mass transport loss in PEMWE. Specifically, the overall cell voltage *E*_cell_ of the PEMWE could be divided into the reversible potential (${E}_{\text{rev}}$), the kinetic overpotential ($\eta_{{{\text{kin}}}}$), the ohmic overpotential ($\eta_{{{\text{ohm}}}}$), and the concentration overpotential ($\eta_{{{\text{mt}}}}$) [206, 221, 222]:

14 $$E_{{{\text{cell}}}} = E_{{{\text{rev}}}} + \eta_{{{\text{kin}}}} + \eta_{{{\text{ohm}}}} + \eta_{{{\text{mt}}}}$$

The ${\text{E}}_{\text{rev}}$ is estimated using the Nernst equation:

15 $$E_{{{\text{rev}}}} = E_{{{\text{rev}}}}^{{0}} + \frac{RT}{{nF}}\ln \left( {\frac{{a({\text{H}}_{{2}} ) \cdot \sqrt {a({\text{O}}_{{2}} )} }}{{a({\text{H}}_{{2}} {\text{O}})}}} \right)$$

where* a*(H_2_), *a*(O_2_), and *a*(H_2_O) are the activity of the H_2_, O_2_, and H_2_O, where *a*(H_2_O) is usually assumed to be 1 as liquid water, and *a*(H_2_) and *a*(O_2_) are their partial pressures divided by the reference pressure. *R* is the ideal gas constant, *T* is the temperature, and *F* is Faraday’s constant. ${\text{E}}_{\text{rev}}^{0}$ is temperature-dependent standard reversible potential. *n* = 2 are the standard potential and the number of electrons transferred. For current conditions, the ${E}_{\text{rev}}^{0}$ is calculated to be 1.168 V obtained from the literature [223] based on the following equation:

16 $$E_{{{\text{rev}}}}^{{0}} = 1.2291 - 0.0008456 \cdot (T - 298.15)$$

The dependence of $\eta_{{{\text{kin}}}}$ and current density *i* is fitted using the Tafel approximation method with iR-corrected cell voltage:

17 $$\eta_{{{\text{kin}}}} = b \times \log_{10} \left( {\frac{i}{{i_{0} }}} \right)$$

where ${b}$ is the Tafel slope, and ${i}_{0}$ is the exchange current density.

$\eta_{{{\text{ohm}}}}$ is calculated by Ohm’s law:

18 $$\eta_{{{\text{ohm}}}} = i \times R_{{{\text{ohm}}}}$$

Finally, mass transport overpotential *η*_mt_ is caused by resistance toward fluid transport across the CL and PTL and ionic transport in the CL.

However, the method to accurately estimate mass transport losses from a polarization curve poses certain challenges [224]. Firstly, it is debated that whether it is reasonable to characterize kinetic losses with a simple Tafel model with iR-corrected cell voltage. The kinetics of the OER are complex, with multiple mechanisms proposed, and the Tafel slope for the OER may change at elevated overpotentials due to the significantly low electronic conductivity—such as that observed in the IrO_x_ catalysts—and the effects of two-phase mass transport due to the evolution of bubbles. A two-dimensional finite element model has been employed by Moore et al. that assumes the presence of a single intermediate species along with two intermediate reactions. This approach aims to mitigate the inaccuracies arising from the complexity associated with the OER [208]. Secondly, the use of the HFR to characterize the ohmic losses may neglect part of the CL resistance and interfacial resistance between the PTL and ACL. The effect of bubbles has been present since the small current densities, while the possible mechanism of the effect of bubbles on the Tafel slope and ohmic loss has not yet been unclear. More accurate electrochemical methods need to be developed in subsequent studies to fully characterize the influence of bubbles in ACLs.

The progress of physical and chemical characterization methods makes it possible to find out how oxygen transport influence overpotential in the ACL from a more microscopic perspective. A high-speed imaging system combined with a transparent reaction-visible cell design could provide a powerful method for bubble visualization in the two-phase transport process [217, 220, 225]. This can realize the observation of undesired bubble behavior in ACLs, so as to facilitate the management of mass transport [220, 225]. The applications of QCM in the study of the formation of nanobubbles are employed by Zhang et al. [226], giving the high sensitivity, fast response, and parallel measurements by using multiple channels. This method could measure the formation of nanobubbles on the crystal surfaces, which can yield easily detectable shifts of frequency and dissipation, rather than measure the bigger bubbles directly in water. Recently, with the rapid development of synchrotron radiation, more in situ characterization methods can be tested down to the molecular scale. For example, in situ Raman was able to further characterize the bubble formation process, and the researchers observed the bubbles in the model microelectrode. However, the current research method is relatively insufficient to characterize the oxygen transport inside ACLs in operando, and the existence of bubbles in ACLs cannot be directly determined.

### Oxygen Transport Mechanism of PEMWEs

**Fig. 13:**
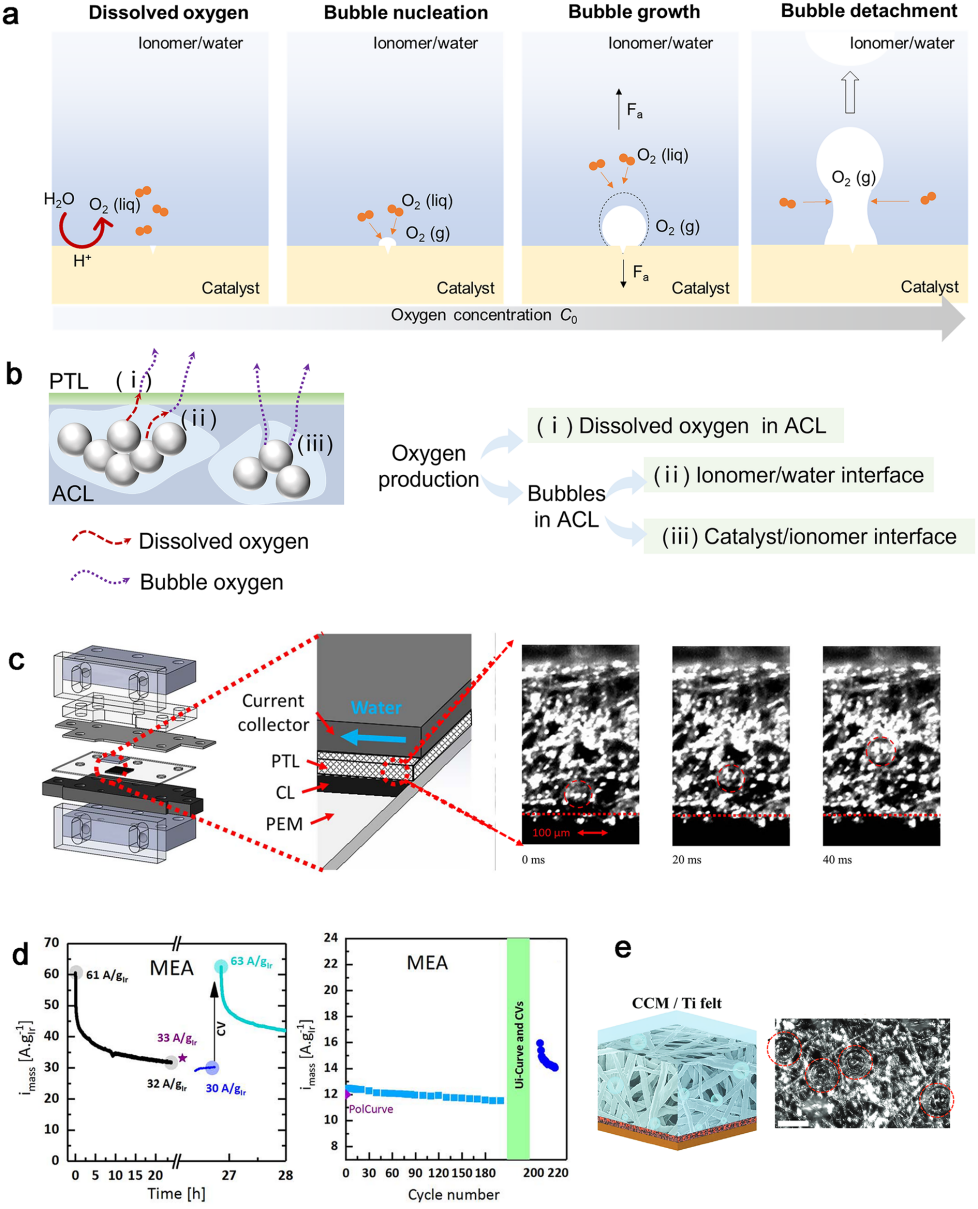
Oxygen transport mechanism in ACLs in PEMWE. **a** Oxygen transport process for dissolved oxygen, oxygen bubbles nucleation, growth, and detachment. **b** Three kinds of possible oxygen transport pathway. **c** Correlation of enlarged schematic diagram and shooting field of view [232]. 2022 The Author(s). **d** Chronoamperometry at 1.53 V vs. RHE (HFR corrected) measured in an MEA [207]. 2021 The Author(s). **e** Schematics of CCM/Ti felt and corresponding high-speed video snapshot [220]. Copyright 1999–2025 John Wiley & Sons, Inc. or related companies

The dissolved or bubble oxygen would appear based on different oxygen concentrations* C*_0_ in the anode PEMWEs. The life cycle of the oxygen evolution typically consists of four stages: dissolved oxygen, bubble nucleation, bubble growth, and bubble detachment (Fig. 13a) [227, 228].

Bubble nucleation commonly described by classical nucleation theory is the stochastic formation of a gas molecules cluster from a solution supersaturated with dissolved gas. It is driven by an increased chemical potential of dissolved gas molecules near the electrode surface. In electrochemical processes, the gas supersaturation of a liquid $\xi$ at a pressure *P* can then be expressed as follows when only a single gas species is present:

19 $$\xi = \frac{{C_{0} - C_{{{\text{sat}}}} }}{{C_{{{\text{sat}}}} }} = \frac{{C_{0} }}{{K_{{\text{H}}} P}} - 1$$

The dissolved oxygen appears when the oxygen concentration *C*_0_ is lower than the equilibrium or saturation concentration of dissolved gas in a liquid, *C*_sat_, which is proportional to the partial pressure *P* of the gas acting on a liquid surface based on Henry’s law. If *ξ* > 0 (*C*_0_ > *C*_sat_), the liquid is said to be supersaturated, and the value of *ξ* could help to decide the tendency to produce bubbles [229]. Nucleation occurs typically on cracks and crevices in the electrode; with the continuous increase in the oxygen concentration, the dissolved oxygen begins to transform into bubble oxygen. The maximus supersaturation values *ξ* for oxygen evolution in PTLs, at which bubble nucleation is experimentally observed, are in the range of 100–1000 [230], which depends on several factors, such as temperature, pressure, current densities, pore structure, and wettability of the electrode and the electrolyte properties.

A bubble then grows with the increased* C*_0_, driving dissolved gas from the liquid to the bubble. The concentration of dissolved gas in the liquid immediately adjacent to the interface of a spherical bubble, *C*_b_, can be obtained by Laplace–Young equation and Henry’s law as follows:

20 $$C_{{\text{b}}} = K_{{\text{H}}} P_{{\text{i}}} = K_{{\text{H}}} \left( {\frac{2\gamma }{r} + P_{0} } \right)$$

Here, *P*_i_ denotes the gas pressure inside the bubble with radius *r*, and *γ* is the surface tension of the gas–liquid interface, which is the energy required to increase the surface of a liquid due to intermolecular interactions. The quantity $\frac{2\gamma }{r}$ is known as the Laplace pressure valid for small bubbles, assuming that there is a constant curvature. When *C*_0_ is higher than *C*_b_, it drives the flux of dissolved gas from the liquid phase to bubbles.

Then bubble attachment is caused by the buoyant force overcoming the adhesion force on a bubble. After detachment, it re-exposes the active area that was previously blocked by attached bubbles. As for the specific content of the mechanism of bubbles in PEMWE, Yuan et al. have carried out a detailed analysis [231].

For ACLs, the evolution process of bubbles is more complicated due to the existence of microscopic aggregates and pore structures in its structure. Oxygen is generated from the catalytic sites and transports from the ionomer agglomeration of ACL, while diffusing in the porous structure of ACL and PTL. The oxygen bubbles rather than the dissolved form in flow field and PTLs have been proved due to the high concentration. However, the mechanisms in ACLs are not clear enough. Whether the nucleation of oxygen bubbles forms in ACLs is still controversial. Depending on where oxygen reaches saturation in the ACL, there exist dissolved oxygen and gaseous oxygen for oxygen in ACL of PEMWE, which should be deeply understand.

Correspondingly, there is a bubble generation site as shown in Fig. 13b: (i) The dissolved oxygen diffuses in both ionomer, water in pore of ACL, and reaches saturation at the CL/PTL interface and produces bubbles; (ii) oxygen is produced in the form of dissolved oxygen in the ionomer covered on catalytic sites, and bubbles are produced at the ionomer/water interface on the ionomer surface; and (iii) oxygen reaches saturation at the catalytic sites, and bubbles are generated at the catalyst/ionomer interface. As shown in Fig. 13c, Watanabe et al. proposed the existence of dissolved oxygen and the formation of an oxygen supersaturated region based on time variation of the bubble radius after the water pump stopped. They observed that oxygen bubbles were generated at the interface between CL/PTL interfaces and believed that the dissolved oxygen concentration decreases with increasing distance from the ACL surface [232]. In this condition, the “ionomer/catalyst/water” TPBs are not restricted by gas phase transport. Dissolved oxygen is generated from the reaction site, and its transport depends on the oxygen transport rate of ionomer and water. On the other hand, some others believed that the gaseous oxygen acts as bubbles in ACLs and then could form a two-phase flow. Tovini et al. conducted a chronoamperometric (CA) aging test as shown in Fig. 13d and found that the current decreased by ∼50% after ∼24 h, suggesting that half of the catalyst is either shielded or has degraded. After scanning the potential to below 1 V vs. RHE by a CA step, the same initial current as that obtained for the first CA step was obtained. This firmly proves that the performance decay during a CA step was solely due to shielding the active sites by oxygen bubbles or the increase in local partial pressure of oxygen [207]. As shown in Fig. 13e, Yu et al. also observed that the oxygen bubbles are accumulated at the reaction interface for the conventional CCM/Ti felt electrode based on the high-speed microscale visualization system [220].

However, the oxygen transport path (ii) or path (iii) of the bubble generation sum is still debated. Suwa et al. presents a visualization study based on electron microscopy with EDX to examine the effect of ionomers on the behavior of oxygen bubbles generated from catalyst covered with ionomers. The oxygen bubbles near the ionomer were clearly observed with a high-speed camera. There were no significant cracks and damages on the ionomers, suggesting that oxygen bubbles are generated on the ionomer surface without an apparent gas interface [233]. This study was conducted at a low current density (1.6 V vs. RHE) and does not fully account for oxygen transport at higher current densities.

In electrochemical processes, nucleation takes place when the concentration of dissolved gas near the electrode surface *C*_0_ becomes large enough [228, 234]. These bubbles are thought to transport through the voids or cracks in the ionomer. Bernt et al. [235] observed that the unassigned voltage losses arise from oxygen transport in ACLs range between ≈ 20 and 25 mV at 3 A cm^−2^ in the MEA with a high ionomer content (28.0 wt%). They hypothesize that for a perfectly crack-free ionomer filling of the anode electrode, the produced oxygen must be removed by permeation through the ionomer phase and required ~ 200 bar between the oxygen pressure in channel *p*_O2, channel_ and the oxygen pressure on catalyst *p*_O2, catalyst_, which is more on the order to the real ACL. Thus, it is believed that the main path for oxygen removal from the electrode is not permeation but convective transport through cracks or pinholes in the ionomer layer within the electrode, as shown in path (iii). In addition to the transport of ionomers and water, the bubbles in the pores of the ACL follow the transport of two-phase flow. The bubble is discharged from ACL by capillary force, and the pore structure and the throat size distributions are important influencing factors.

With the increase in the reaction process or the increase in the current density, the oxygen concentration at the reaction interface increases. Under different current density and electrode structure conditions, the amount of oxygen generated by the electrode is different, and it is reasonable to reserve a discussion of the three-way coupled bubble transport process, but further research is still needed.

### Insight and Challenge in ACLs of PEMWEs

Since the importance of oxygen transport in the PEMWE was recognized and inspired by the low or ultra-low Pt loading PEMFCs, researches for the oxygen transport in the ACLs are divided into the transport in the pore structure and in the ionomer in ACLs, corresponding to the bulk and local oxygen transport, respectively. The strategies for improving oxygen transport primarily focus on enhancing oxygen bubble evolution and dissolved oxygen extraction in ACLs, because they make it difficult for reactant water to reach the catalytic sites, and the properties of TPBs with bubbles including surface forces and adsorption behaviors can be altered.

**Fig. 14:**
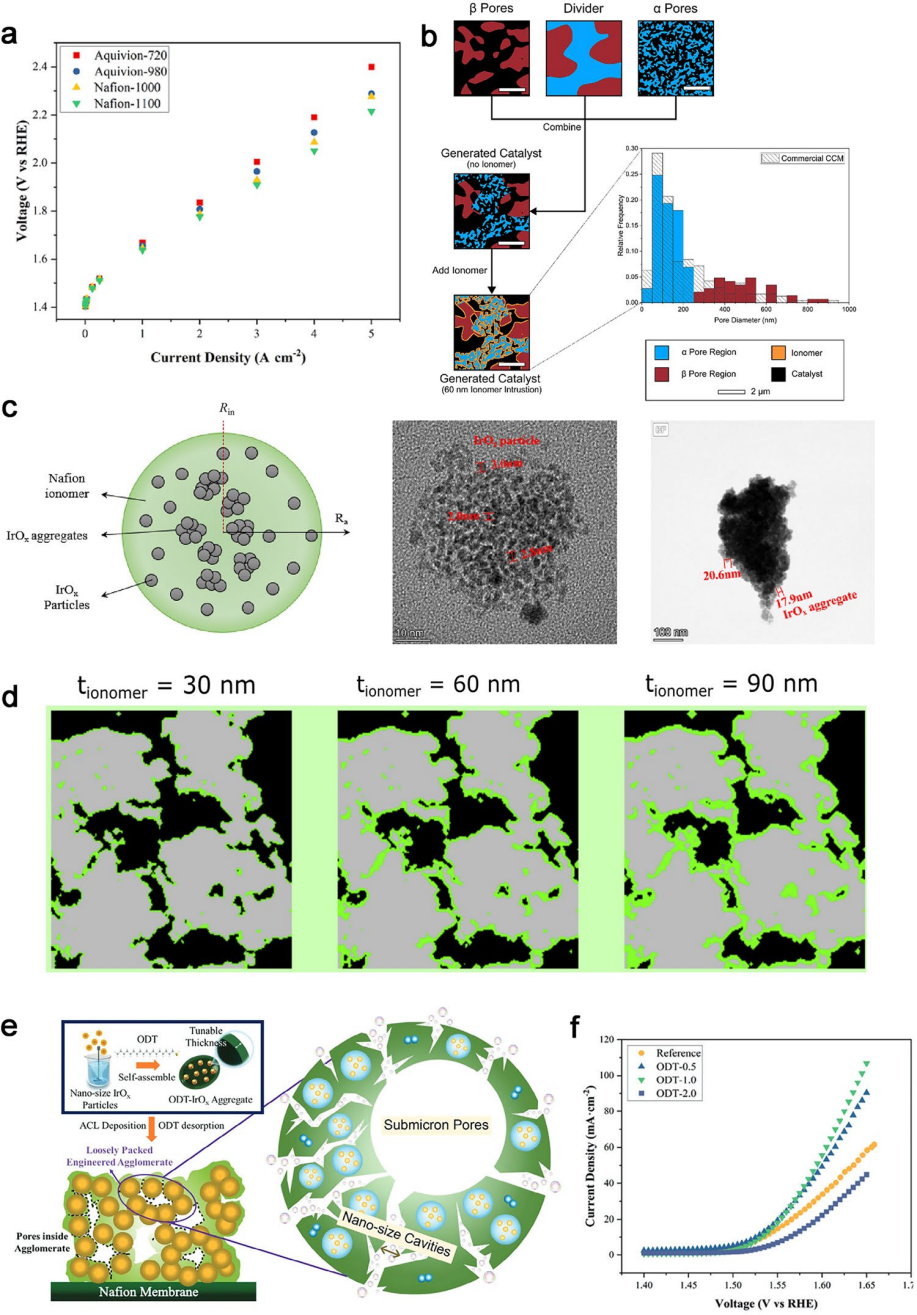
Oxygen transport in pore structure and ionomer in PEMWEs.** a** Polarization curves and mass transport overpotential of MEAs prepared by different ionomers [236]. Copyright 2023 Elsevier B.V. **b** The pore size distribution of the ACL, where the α pores are indicated in blue, the β pores in red, the ionomer in orange, and the iridium oxide catalyst in black [238]; Copyright 2023 Published by Elsevier B.V. **c** Scheme of the catalyst–ionomer agglomerate (left), TEM image of IrO_x_ catalyst particles, and STEM image of an agglomerate [242]. Copyright 2024 American Chemical Society. **d** Cross-sectional images at 50% of the thickness of the iridium-based catalyst with ionomer thicknesses of 30, 60, and 90 nm [239]. Open access. **e** Schematic diagram of the fabrication process for the electrode using agglomerate engineering, and **f** the corresponding LSV curves of electrodes made by ODT [244]. Copyright 1999–2025 John Wiley & Sons, Inc. or related companies

In the presence of only dissolved oxygen at low current densities, oxygen is transported in the ionomer and water in the pores. Inspired by PEMFC, it is necessary to improve oxygen transport in the ionomer in ACLs as follows: (i) reduce the transport path by reducing the content of ionomers or regulating the uniform distribution of ionomers; (ii) increase the oxygen transport capacity of the ionomer, such as the use of HOPI ionomer. Zhao et al. [236] have investigated the influence of ionomers’ EWs and side-chain length on the property of PEMWE ACL and cell performance. The mass transport overpotential is shown in Fig. 14a. It is noted that as the current density increased, the mass transport overpotential increased. To the authors’ knowledge, as the current density increases, more oxygen molecules not yet transported away from ACLs are accumulated around the active reaction sites, leading to a rapid increase in oxygen concentration. Although the ACLs prepared by different PFSA ionomers show similar microstructure, the mass transport overpotential decreases with the increase in EWs in PFSA ionomers under the same current densities. Since HOPI is widely used in PEMFC, as mentioned in Sect. 3.3, it is possible to try to apply HOPI in PEMWE in future work. Under the condition that proton conduction and electron conduction in interface contact are not affected, it is expected to increase the oxygen transport in ionomer and reduce the oxygen concentration at the catalytic site. However, there are few researches to analyze the work in this area. When the current density is high and bubble oxygen is formed, the transport properties of bubbles in the ionomer are very important, and the rapid expulsion of bubbles can ensure the smooth reaction at the catalytic site. Generally, small and uniform bubbles are desired, meaning that the gas generated by the reaction could be removed rapidly and water could be supplied timely.

The pore structure within ACLs plays an important role in the oxygen transport. In fuel cells, the primary pore (< 30 nm) and secondary pore (30–150 nm) have been widely accepted, where the primary pores are between carbon particles inside the agglomerates and secondary pores are the void volumes between the agglomerates [45, 237]. In PEMWEs, the pore size distribution of the commercial IrO_2_ catalysts is comprised of two regions: *α* pore regions (defined as regions with pores < 250 nm in size) and *β* pore regions (defined as regions with pores > 250 nm in size), as shown in Fig. 14b [238]. The disordered distribution of catalyst particles and ionomers results in agglomeration and pore structures of different sizes in the ACL. 3D pore structure of the Ir-based ACLs was investigated by Lee et al. [239] based on the synchrotron full-field transmission X-ray microscopy (TXM), and the reconstruction of the iridium-based catalyst sample proved the existence of pores ranging from tens of nanometers to a few microns in the CL. Both pores at the tens of nanometers and at a few microns in diameter filled with water contribute to catalyst utilization. The oxygen mainly occurs in the macropores of the CL since macropores play a key role in permeability. Corresponding to the dissolved oxygen and bubble oxygen for analysis, oxygen transport mechanism in pore structure is different. For dissolved oxygen, the primary transport paths in the ACL are through the ionomer and water. Notably, the solubility of oxygen in water is approximately four times higher than that in the ionomer. For bubble oxygen, the gas transport in the pore structure is a two-phase flow transport, and the discharge mainly depends on the capillary pressure. Although the impact of two-phase flow, mass transport behavior, and permeability of the PTL has been extensively characterized, characterization of CL two-phase permeability is critically lacking. Pore-scale models are necessary to elucidate the transport mechanisms that occur in the ACL, as they provide microstructure specific information that is difficult to obtain using continuum modeling methods. Lessons from PEMFCs indicate that the pore forming for the CLs would effectively improve the CL’s porosity. Inspired by this, Lv et al. prepared ACLs with different pore size distributions by using the inks containing different CuO particle sizes and obtained the optimal electrocatalytic performance (2.043 V @ 3 A cm^−2^), which is lower than 163 mV than that of ACL without pore-forming treatment due to enhanced transport properties [240]. MIP results demonstrate that the porosity increases from 38.8% to 52.3% after the pore-forming treatment. Mandal et al. added carbon to the Ir catalyst ink, which is then oxidized in situ, and the CL porosity can be increased from 58% to 77% while the number of CL cracks is decreased [241]. The selection of an optimal pore-forming agent that effectively balances proton and mass transport is essential for the structural optimization of the PEMWE in order to enhance their overall performance.

Instead of coating on the carbon and catalyst surface in a form of thin film, as in a CCLs in PEMFCs, the ionomer forms catalyst–ionomer agglomerate in PEMWE due to encapsulation of the small catalyst particles by the ionomer, which also suggests the important role of ionomers in mass transport in ACLs. Specific morphologies of catalyst–ionomer agglomerates were characterized by Yuan et al. using STEM as shown in Fig. 14c. Upon dispersion of IrO_*x*_ catalysts nanoparticles with the size of 2–3 nm in the solution, agglomerates are formed with an average size in the size range of 193–247 nm due to the different type of catalysts [239, 242]. The ionomer serves as a vital role to the proton and mass transport during the OER, and the ionomer content is an essential impact factor for the morphology of catalyst–ionomer agglomerate via pore structure in ACLs [235, 239]. Although the ionomer could separate the bubble evolution sites from the OER sites and hinder the instant deactivation caused by the bubble coverage, a gradual performance loss could also be caused by local water starvation and deteriorate the catalyst performance due to the affinity of the ionomer surface for bubbles [242]. The oxygen bubbles transport in catalyst–ionomer agglomerate depends deeply on pore structure in ACLs. Combining the cross-section images from synchrotron TXM and pore network modeling, Lee et al. observed ionomer layers with thicknesses of 30, 60, and 90 nm (Fig. 14d), which described for low, medium, and high ionomer content, respectively. It could be concluded that for high ionomer contents, performance losses can be related to high oxygen transport resistance resulting from a filling of the electrode void pore by ionomer. The electronic contact resistance and the electronic insulation of the ionomer also increased in high ionomer content. For low ionomer content, the proton conduction resistance could be high [239]. It is necessary to achieve a balance between oxygen bubbles transport and proton conduction catalyst–ionomer agglomerate through optimizing ionomer content or introducing additional transport paths into the catalyst–ionomer agglomerate. The ionomer-free ACLs at ultra-low loadings (< 0.1 mg_Ir_ cm^−2^) with a thin-film catalyst layer were also fabricated by Lee et al., and the electrode exhibited lower mass transport overpotentials compared to the conventional ionomer Ir PTE especially at high current densities. It is noted that the thin film from CL could not be limited by proton transport and thus could be fabricated without ionomer, but, anyway, this demonstrates the importance of ionomer for quality transmission and the importance of ionomer content [222].

In addition, the densely packed ionomer structure at the TPBs leads to high oxygen transport resistance, which slows the transport of the generated oxygen to the free bulk liquid phase and thus increases transport resistance. The ionomer network blocking could be reduced as the extended ion transport paths and eventually improves the mass transport. Therefore, the TPBs regulation based on catalyst–ionomer agglomerate to enhance the dissolved oxygen and bubble oxygen transport process inside the agglomerate should be warranted more attention. The random distribution of catalysts and ionomers usually makes it hard to achieve sufficient pores with appropriate size and porosity. Therefore, methods for agglomerate engineering should be proposed for boosting PEMWE performance. As shown in Fig. 14e, f, Zhao et al. used 1-octadecanethiol (ODT, one of the commonly used thiols) as the physical barrier to suppress the dense packing of IrO_*x*_ particles and aggregates. It inhibits excessive aggregation and generates a profusion of submicron pores and nanocavities within the agglomerate and eventually the improved electrolysis current density approaches 7.0 A cm^−2^ at 2.07 V, leading to an increase in catalyst utilization up to 0.078 g_Ir_ kW^−1^. Another effective way to enhance water electrolysis efficiency by bubble management is to increase the hydrophilic surfaces in the catalyst–ionomer agglomerate. The wettability characteristics exhibit contrasting behaviors in the gas and liquid phases. Specifically, hydrophobic surfaces demonstrate aerophilicity, whereas hydrophilic surfaces display aerophobia when immersed in aqueous media. Hydrophilicity can be controlled effectively by adding hydrophilic substances to the electrode structure. For example, polyethylene glycol (PEG) was utilized as a slurry additive for ACLs by Wang et al. to control the wettability. The CL contact angle decreased from 139.8° to 72.3°, leading the performance of the WE which can reach 3.07 A cm^−2^@1.9 V higher than that of DOE 2025 target [243].

## Conclusion and Outlook

Recent developments in the hydrogen economy have underscored the necessity for efficient hydrogen production and utilization. Excess energy generated from renewable sources such as solar or wind sources can be stored through water electrolysis, subsequently transported to various regions, and utilized in fuel cells to facilitate the interconversion of the hydrogen and electricity. The PEMWE and PEMFC technologies serve as key components in the upstream and downstream segments of the hydrogen industry, respectively, and have garnered significant attention recently. Considerable effort has been devoted to minimize the utilization of precious metals used as electrocatalysts, such as Pt in PEMFC and Ir/IrO_x_ in PEMWE, or replace them with nonprecious catalysts. Given that the CLs encompass intricate pore structures and interfaces involving gases (H_2_ and O_2_), liquids (water or/and electrolytes), and solids (catalysts), the oxygen transport behavior ultimately hinders the PEMWE and PEMFC from achieving high current densities and realizing commercial success. This paper addresses the issues of oxygen transport within the CLs of PEM hydrogen and electricity conversion devices, including the PEMFC and PEMWE, and the performance improvement strategies of the devices has been proposed.

Firstly, oxygen transport mechanisms in the CCL of PEMFCs are reviewed. The bulk oxygen transport resistance is mainly affected by pore structure properties, and the local one is considered be affected by the structure and morphology of ionomer films at the TPB. The number of catalytic sites is reduced under low or ultralow Pt loading conditions, and the corespondingly microscale increase of the local oxygen tranpsort routes at the TPB greatly affects fuel cell performance in high current density. We proposed that the regulation of TPBs will be the focus of subsequent research in terms of the optimization of electrode pore structure, carbon support property and structure, as well as ionomer distribution and structure.

The systematically review of PEMFCs has significant implications for oxygen transport in PEMWE in terms of the comparable MEA structure. At present, there are still some controversies about the existence forms of oxygen in the ACL of PEMWEs, but we concluded from previous studies that there exists in the form of dissolved oxygen at low current density and in the form of bubble oxygen at high current density. The rapid removal of oxygen in the ACL could prevent the oxygen bubbles from affecting the transport path of reactant water. For the transport in the pore structure, dissolved oxygen is transferred from water, and bubble oxygen is transferred in the form of two-phase flow in water. Increasing the pore size is conducive to both types of sample transport. For oxygen transport in the ionomer on the catalyst surface, dissolved oxygen depends on the oxygen transport capacity of the ionomer. Therefore, ionomer with high oxygen transport properties should be designed to enhance oxygen transport and adjust the distribution of the ionomer at the same time. For bubble oxygen, it could transport through the crack of the ionomer–catalyst aggregates, so the effective bubble transport channel in the ionomer can increase the bubble transport. This resembles the more advanced and diversified pore structure and interface adjustment mechanism found in PEMFCs, thus offering certain inspiration.
